# mRNA-1273 vaccination protects against SARS-CoV-2–elicited lung inflammation in nonhuman primates

**DOI:** 10.1172/jci.insight.160039

**Published:** 2022-07-08

**Authors:** Adam T. Waickman, Kaitlin Victor, Krista Newell, Tao Li, Heather Friberg, Kathryn E. Foulds, Mario Roederer, Diane L. Bolton, Jeffrey R. Currier, Robert Seder

**Affiliations:** 1 Department of Microbiology and Immunology and; 2 Institute for Global Health and Translational Sciences, State University of New York Upstate Medical University, Syracuse, New York, USA.; 3 Viral Diseases Branch, Walter Reed Army Institute of Research, Silver Spring, Maryland, USA.; 4 Vaccine Research Center, National Institute of Allergy and Infectious Diseases, NIH, Bethesda, Maryland, USA.; 5 US Military HIV Research Program, Walter Reed Army Institute of Research, Silver Spring, Maryland, USA.; 6 Henry M. Jackson Foundation for the Advancement of Military Medicine, Bethesda, Maryland, USA.

**Keywords:** COVID-19, Immunology, Cellular immune response

## Abstract

Vaccine-elicited SARS-CoV-2 antibody responses are an established correlate of protection against viral infection in humans and nonhuman primates. However, it is less clear that vaccine-induced immunity is able to limit infection-elicited inflammation in the lower respiratory tract. To assess this, we collected bronchoalveolar lavage fluid samples after SARS-CoV-2 strain USA-WA1/2020 challenge from rhesus macaques vaccinated with mRNA-1273 in a dose-reduction study. Single-cell transcriptomic profiling revealed a broad cellular landscape 48 hours after challenge, with distinct inflammatory signatures that correlated with viral RNA burden in the lower respiratory tract. These inflammatory signatures included phagocyte-restricted expression of chemokines, such as *CXCL10* and *CCL3*, and the broad expression of IFN-induced genes, such as *MX1*, *ISG15*, and *IFIT1*. Induction of these inflammatory profiles was suppressed by prior mRNA-1273 vaccination in a dose-dependent manner and negatively correlated with prechallenge serum and lung antibody titers against SARS-CoV-2 spike. These observations were replicated and validated in a second independent macaque challenge study using the B.1.351/Beta variant of SARS-CoV-2. These data support a model wherein vaccine-elicited antibody responses restrict viral replication following SARS-CoV-2 exposure, including limiting viral dissemination to the lower respiratory tract and infection-mediated inflammation and pathogenesis.

## Introduction

Severe acute respiratory syndrome coronavirus 2 (SARS-CoV-2) — the causative agent of COVID-19 — has infected at least 250 million individuals and resulted in over 5 million deaths as of November 2021 (1). SARS-CoV-2 infection results in a range of clinical outcomes, from asymptomatic clearance to severe lung pathology with concomitant acute respiratory distress. Almost all of the morbidity and mortality attributable to SARS-CoV-2 is seen in the minority of patients who develop severe pneumonia requiring mechanical ventilation (2, 3). This has led to speculation that SARS-CoV-2 infection may promote a unique pathophysiology in which dysregulated immune responses to infection in the lower respiratory tract augment the severity of COVID-19. Indeed, examinations of the cellular composition of bronchoalveolar lavage fluid (BALF) from acutely ill patients with COVID-19 have revealed a cellular landscape containing both resident cells and infiltrating immune cells displaying a unique and dysregulated inflammatory profile (4). Taken together, these data are consistent with a model wherein SARS-CoV-2–infected cells engage in a positive feedback loop with infiltrating immune cells to potentiate persistent alveolar inflammation and pathology (5). An effective vaccine would then be expected to impede the initiation of this, or a similar, pathological feedback loop, thereby limiting lower airway disease.

Single-cell RNA sequencing (scRNA-Seq) is a highly sensitive tool for analyzing the spectrum of SARS-CoV-2–elicited inflammation and the impact of vaccine-mediated immunity. The use of this approach to examine human BALF and PBMC samples has already identified several populations of immune cells likely implicated in inflammation-driven immunopathology and vaccine-mediated protection as well as those likely to contain SARS-CoV-2 genetic material (5–10). Studies of human PBMCs using scRNA-Seq have demonstrated a dysregulated response in both innate and adaptive immune cells in severe disease (11), evidence of emergency myelopoiesis and neutrophil dysregulation in severe disease (6), and an upregulation of the TNF/IL-1β-driven inflammatory response as compared with that in influenza in classical monocytes (12). The unifying theme of these studies is that in severe COVID-19, compared with mild disease or asymptomatic infection, there is a profound and dysregulated type I IFN response across many lymphoid and myeloid origin cells (13). This response is accompanied by hyperinflammation, evidence of cellular proliferation, and defective antigen presentation and IFN responsiveness in classical monocytes.

Animal models have recapitulated many key aspects of the inflammatory response observed in the human lung, such as viral shedding, cellular infiltration profiles, and cellular inflammatory profiles at the transcriptional level (14, 15). These models provide the critical ability to control dose and exposure variables that present a fundamental barrier to the accurate interpretation of human studies. Additionally, rhesus macaques (*Macaca mulatta*) represent a clinically relevant model for assessing lung tissue pathology and temporal analysis of SARS-CoV-2–elicited inflammation, and they are the preclinical gold standard for assessing SARS-CoV-2 vaccine efficacy (16–22). Treatment of rhesus macaques with a clinically approved JAK1/JAK2 inhibitor resulted in reduced lung inflammation and pathology, corresponding with attenuated infiltration of inflammatory immune cells and NETosis (15). These outcomes were associated with suppression of neutrophil recruitment and production of cytokines and chemokines by inflammatory macrophages, despite comparable type I IFN responses. Similarly, responses to SARS-CoV-2 in ferrets revealed a shift in BALF macrophage gene expression signatures toward a proinflammatory phenotype during early infection (23), underscoring the critical need to understand the cellular complexities of SARS-CoV-2–elicited inflammation.

mRNA-based vaccine platforms — such as Moderna’s mRNA-1273 and Pfizer/BioNTech’s BNT162b2, which encode a stabilized version of the SARS-CoV-2 spike glycoprotein (24) — show more than 90% efficacy against symptomatic COVID-19 in initial phase III analyses and in large-scale prospective studies performed after their global rollout (25, 26). However, the efficacy of these vaccines against severe lower airway disease wanes over time after the initial prime and boost (27–29). Preclinical and clinical studies have strongly suggested that vaccine-elicited serum levels of SARS-CoV-2–neutralizing antibody titers are a mechanistic immune correlate of vaccine efficacy (30, 31). Despite the abundance of clinical and preclinical efficacy data for these mRNA-based vaccine platforms, there is little prospective information currently available on how these vaccines impact SARS-CoV-2–elicited inflammation in the lower respiratory tract with any degree of spatial or temporal resolution.

In this study, we sought to understand the effects of mRNA-1273 vaccination on the cellular inflammatory response to SARS-CoV-2 infection in the lower respiratory tract of nonhuman primates (NHPs) and whether vaccination is capable of breaking the inflammatory feedback loop that characterizes severe COVID-19. We used scRNA-Seq to analyze BALF cells from rhesus macaques challenged with SARS-CoV-2 strain USA-WA1/2020 after vaccination with 2 doses of 30 μg or 1 μg mRNA-1273 or PBS. mRNA-1273 vaccination limited SARS-CoV-2–elicited inflammation in the lower respiratory tract, as defined by the expression of proinflammatory chemokines and cytokines in multiple cell types as well as the broad reduction in expression of IFN gene products, such as *MX1*, *ISG15*, and *IFIT1*. Additionally, SARS-CoV-2–elicited inflammation was directly associated with postchallenge viral titers and inversely associated with prechallenge antibody levels in unvaccinated and mRNA-1273–vaccinated animals. The ability of mRNA-1273 to limit SARS-CoV-2–elicited inflammation in the lower respiratory tract was independently verified using the antigenically disparate B.1.351/Beta variant. Collectively, these results demonstrate that vaccination with mRNA-1273 not only limits SARS-CoV-2 viral replication, but restricts inflammation in NHPs. Additionally, these data support a model wherein neutralizing antibody at the site of virus inoculation reduces the viral burden, constraining upper respiratory tract viral replication and secondary viral dissemination to the lower respiratory tract and infection-associated inflammation.

## Results

### Frequency of BALF-resident cells following SARS-CoV-2 challenge.

It has previously been demonstrated that vaccination of macaques with mRNA-1273 results in robust serum antibody responses and high-level protection from subsequent SARS-CoV-2 challenge in a dose-dependent fashion (19, 30). To extend these observations and to assess the effect of vaccination on SARS-CoV-2–elicited inflammation in the lower respiratory tract, we performed scRNA-Seq analysis of fresh BALF obtained on days 2 and 7 after SARS-CoV-2 challenge in animals that previously received either 30 μg (*n =* 4) or 1 μg (*n =* 6) mRNA-1273 in a prime-boost series administered 4 weeks apart. Control animals received PBS (*n =* 6). All animals were challenged i.n./intratracheally (i.n./i.t.) with 8 × 10^5^ PFU of SARS-CoV-2 (strain USA-WA1/2020) 4 weeks after the last vaccine dose. In addition, BALF cells were analyzed from naive uninfected animals to serve as controls.

**Figure 1:**
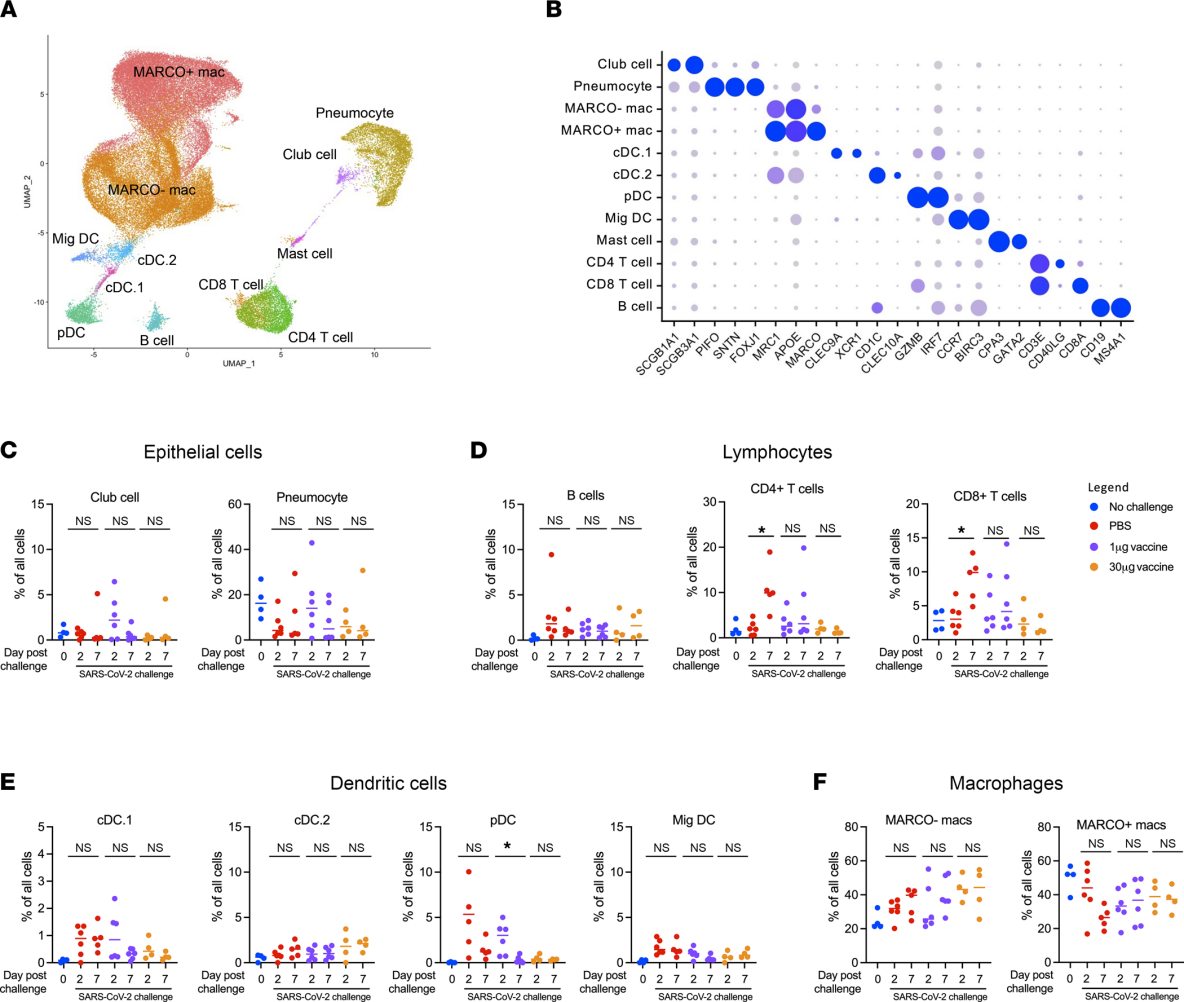
Identification and quantification of BALF cells by scRNA-Seq. (**A**) UMAP projection of BALF cells captured by scRNA-Seq analysis. (**B**) Expression of key linage-specific genes in all annotated cell types. (**C**) Frequency of epithelial cell populations. (**D**) Frequency of lymphocyte cell populations. (**E**) Frequency of dendritic cell populations. (**F**) Frequency of macrophage populations. *P < 0.05, paired *t* test.

scRNA-Seq was used to classify and quantify the cell composition and dynamics within the BALF after challenge. A total of 65,226 viable and high quality BALF cells from all animals were recovered after filtering and quality control steps (Figure 1, A and B). Of note, epithelial cells (Figure 1C), lymphocytes (Figure 1D), dendritic cells (Figure 1E), and macrophages (Figure 1F) were identified at all time points from all animals. Alveolar macrophages were further separated into either MARCO^–^ or MARCO^+^ populations, corresponding to interstitial and tissue-resident alveolar macrophages, respectively (14, 32). Following SARS-CoV-2 challenge, CD4^+^ and CD8^+^ T cells increased in frequency between days 2 and 7 after challenge in unvaccinated animals. Several DC populations also trended higher among unvaccinated infected animals at 1 or more time points relative to uninfected controls. No significant changes were observed in the frequency of epithelial cell or macrophage populations.

### Inflammatory signatures of SARS-CoV-2 infection.

**Figure 2:**
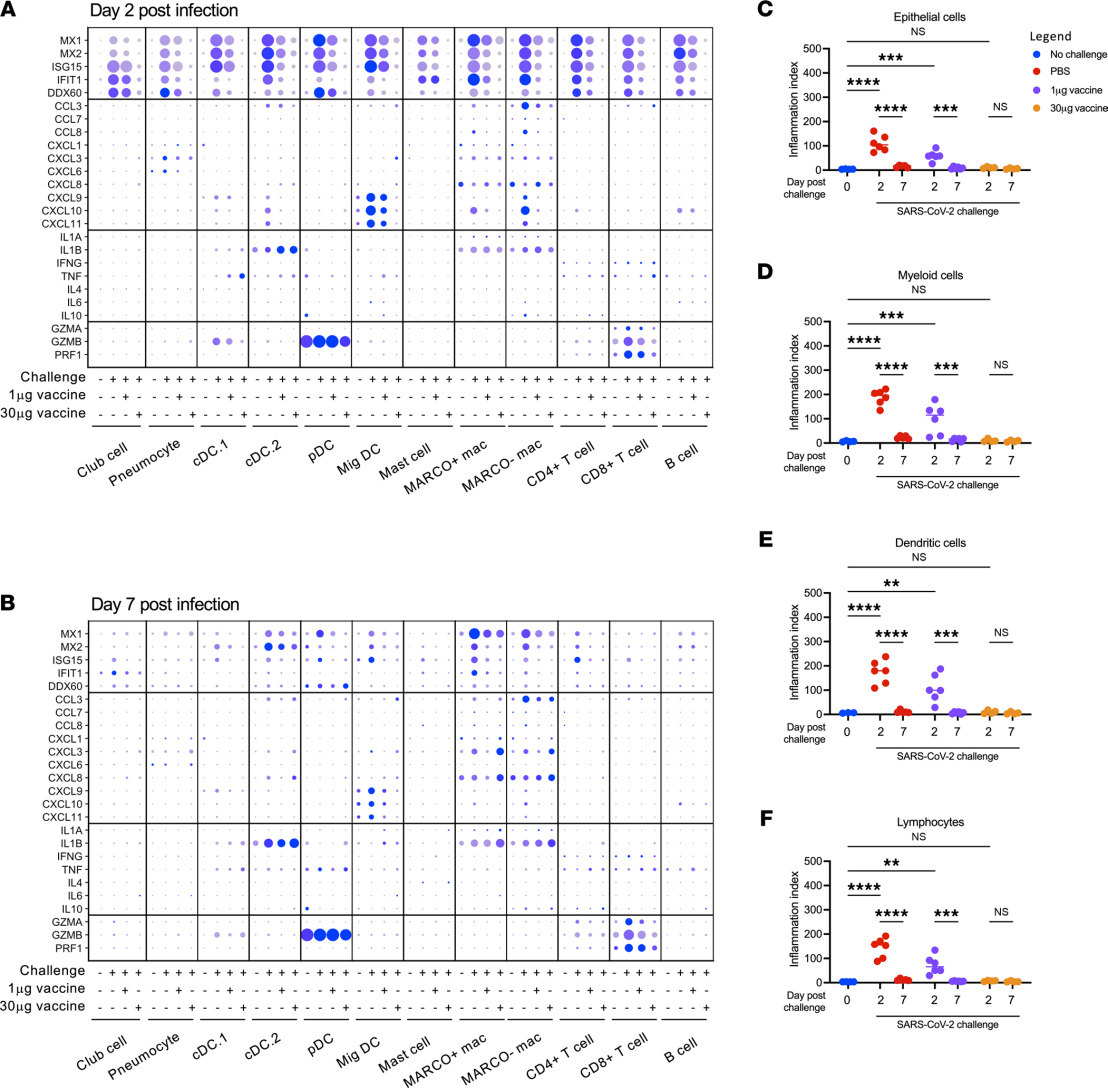
Transcriptional signatures of SARS-CoV-2 WA-1–elicited inflammation. (**A**) Expression of inflammatory markers and cytokines/chemokines in all annotated cells 2 days after infection. (**B**) Expression of inflammatory markers and cytokines/chemokines in all annotated cells 7 days after infection. (**C**) Inflammatory index scores in epithelial cells. (**D**) Inflammatory index scores in myeloid cells. (**E**) Inflammatory index scores in dendritic cells. (**F**) Inflammatory index scores in lymphocytes. **P < 0.01, ***P < 0.001, ****P < 0.0001, 1-way ANOVA with correction for multiple comparisons.

To assess the inflammatory response elicited by SARS-CoV-2 challenge in naive and mRNA-1273–vaccinated animals we performed differential gene expression analysis comparing the transcriptional profile among the experimental study groups across all annotated cell types (Supplemental Figure 1 and Supplemental Data Set 1; supplemental material available online with this article; https://doi.org/10.1172/jci.insight.160039DS1). An inflammatory/type I IFN driven response to infection — as indicated by the expression of genes such as *MX1*, *ISG15*, and *IFIT1* — was observed across all cell types in the unvaccinated and infected animals on day 2 after SARS-CoV-2 challenge relative to both naive animals and animals vaccinated with 30 μg mRNA-1273 (Figure 2A). Gene network analysis performed using Ingenuity Pathway Analysis (IPA; QIAGEN) revealed that BALF cells from unvaccinated and infected animals displayed coordinated gene expression profiles, constant with acute RNA virus infection relative to cells isolated from naive animals or previously vaccinated animals following SARS-CoV-2 challenge (Supplemental Tables 1–8 and Supplemental Data Set 2). The Ingenuity pathways preferentially expressed in unvaccinated and SARS-CoV-2–infected animals relative to uninfected or vaccinated and infected animals include coronavirus pathogenesis pathways and IFN signaling pathways, while pathways associated with protein translation and elongation (EIF2 signaling and p70S6K signaling) were suppressed (Supplemental Tables 1–8, Supplemental Figures 2 and 3, and Supplemental Data Set 2).

Expression of these inflammatory gene markers decreased in a dose-dependent fashion in animals vaccinated with 1 μg or 30 μg mRNA-1273 (Figure 2A). These transcriptional signatures of acute viral infection resolved in most cell types by day 7 after infection, with the exception of lingering *MX1*/*MX2* expression in some populations of macrophages and DCs (Figure 2B). Migratory DCs and MARCO^–^ macrophages responded to SARS-CoV-2 challenge in unvaccinated animals by expressing chemokines such as *CXCL10* (also known as IP10) an *CCL3* (also known as MIP-1A), both of which were previously identified in the context of acute SARS-CoV-2 infection in humans (4). In addition, elevated expression of cytotoxic factors *GZMA* and *PRF1* was observed in CD8^+^ T cells following SARS-CoV-2 challenge on day 2 and maintained 7 days after challenge. Notably, the expression of these proinflammatory chemokines and chemokines was dramatically suppressed in vaccinated animals in a dose-dependent manner across all time points.

To reduce the complexity of the data and provide more direct insight into the dynamics of SARS-CoV-2–elicited inflammation, we defined a transcriptional “inflammation index” that could be used to quantify the level of enrichment for inflammatory gene products in a given sample and cell type. This index was developed by selecting 8 genes (*MX1*, *MX2*, *IFIT1*, *IFIT2*, *IFIT3*, *IFI6*, *ISG15*, and *ISG20*) that were (a) previously known to be regulated at a transcriptional level by viral infection and/or IFN stimulation, (b) highly induced in our data set following SARS-CoV-2 challenge, and (c) consistently observed in all cell types captured in our analysis. Using this reductionist approach, we observed a dose-dependent suppression of SARS-CoV-2–elicited inflammation in epithelial cells (pneumocytes, club cells), myeloid cells (MARCO^+^ macrophages, MARCO^–^ macrophages, mast cells), dendritic cells (conventional dendritic cells 1 [cDC.1], cDC.2, plasmacytoid dendritic cells [pDCs], and migratory dendritic cell [MigDCs]), and lymphocytes (B cells, CD8^+^ T cells, CD4^+^ T cells) with increasing mRNA vaccination dose (Figure 2, C–F). Furthermore, inflammation in animals that received the full dose of vaccine was nearly equivalent to that of the unchallenged control animals in all cell types assessed. Inflammation returned to baseline in all groups by day 7 after vaccination. These results establish a single metric for quantifying the transient inflammatory transcriptional response elicited following SARS-CoV-2 infection across multiple cell populations using scRNA-Seq and by extension provide a measurement of the site-specific host-response to the virus.

### Cell-associated viral RNA burden following SARS-CoV-2 infection.

**Figure 3:**
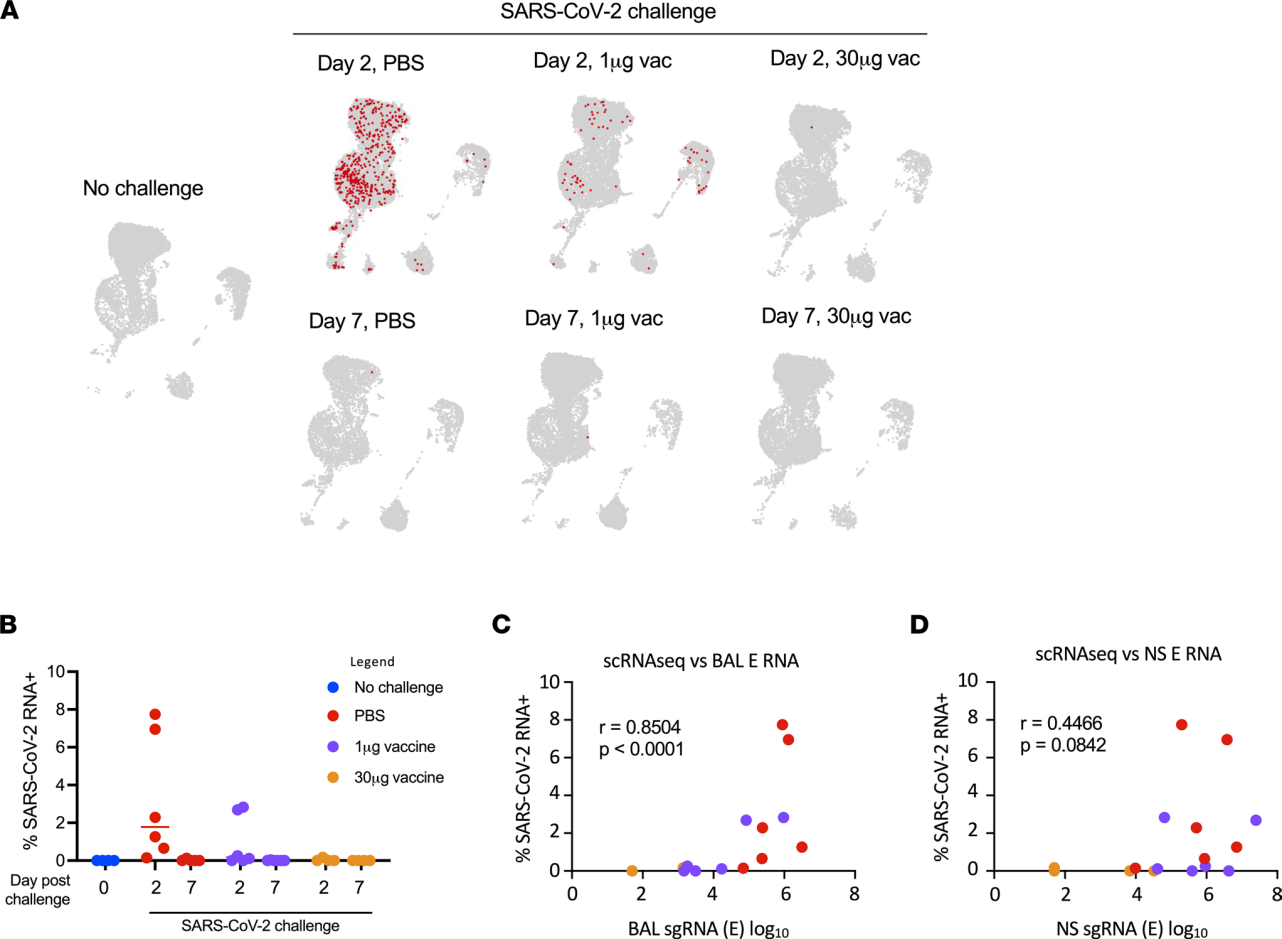
Identification and quantification of SARS-CoV-2 RNA^+^ cells. (**A**) Location of SARS-CoV-2 RNA^+^ cells. (**B**) Frequency of SARS-CoV-2 RNA^+^ cells. (**C**) Relationship between BALF RNA load and frequency of SARS-CoV-2 RNA^+^ cells. (**D**) Relationship between NS RNA load and frequency of SARS-CoV-2 RNA^+^ cells. Spearman’s correlation was used to assess significance.

Having defined the effect of mRNA-1273 vaccination on SARS-CoV-2–associated inflammation in the lower respiratory tract of macaques, we next attempted to quantify the cell-associated SARS-CoV-2 viral RNA burden by aligning scRNA-Seq reads that failed to align to the macaque genome against the SARS-CoV-2 USA-WA1/2020 reference genome. BALF contained widespread SARS-CoV-2 RNA^+^ cells on day 2 in unvaccinated animals (Figure 3, A and B). SARS-CoV-2 RNA^+^ cells were seen in all annotated cell types, with the exception of mast cells in unvaccinated animals, although the greatest number of viral RNA^+^ cells were found in the MARCO^–^ macrophage cluster. Similar to the inflammation index, the frequency of viral RNA^+^ cells was suppressed by vaccination in a dose-dependent fashion and mostly resolved by day 7 after infection. The frequency of viral RNA^+^ cells in the BALF on day 2 correlated well with contemporaneous viral subgenomic RNA (sgRNA) load in the BALF, as quantified by PCR of the E and N gene (Figure 3C and Supplemental Figure 4). Notably, the correlation between the frequency of viral RNA^+^ cells in the BALF with the upper respiratory tract (nasopharyngeal swab) sgRNA loads was weaker (Figure 3D and Supplemental Figure 4). These results show that SARS-CoV-2 viral burden in lung cells is abrogated by mRNA vaccination and is consistent with reduced soluble viral RNA measures in BALF.

### Relationship between SARS-CoV-2 RNA load and cell type–specific inflammation.

**Figure 4:**
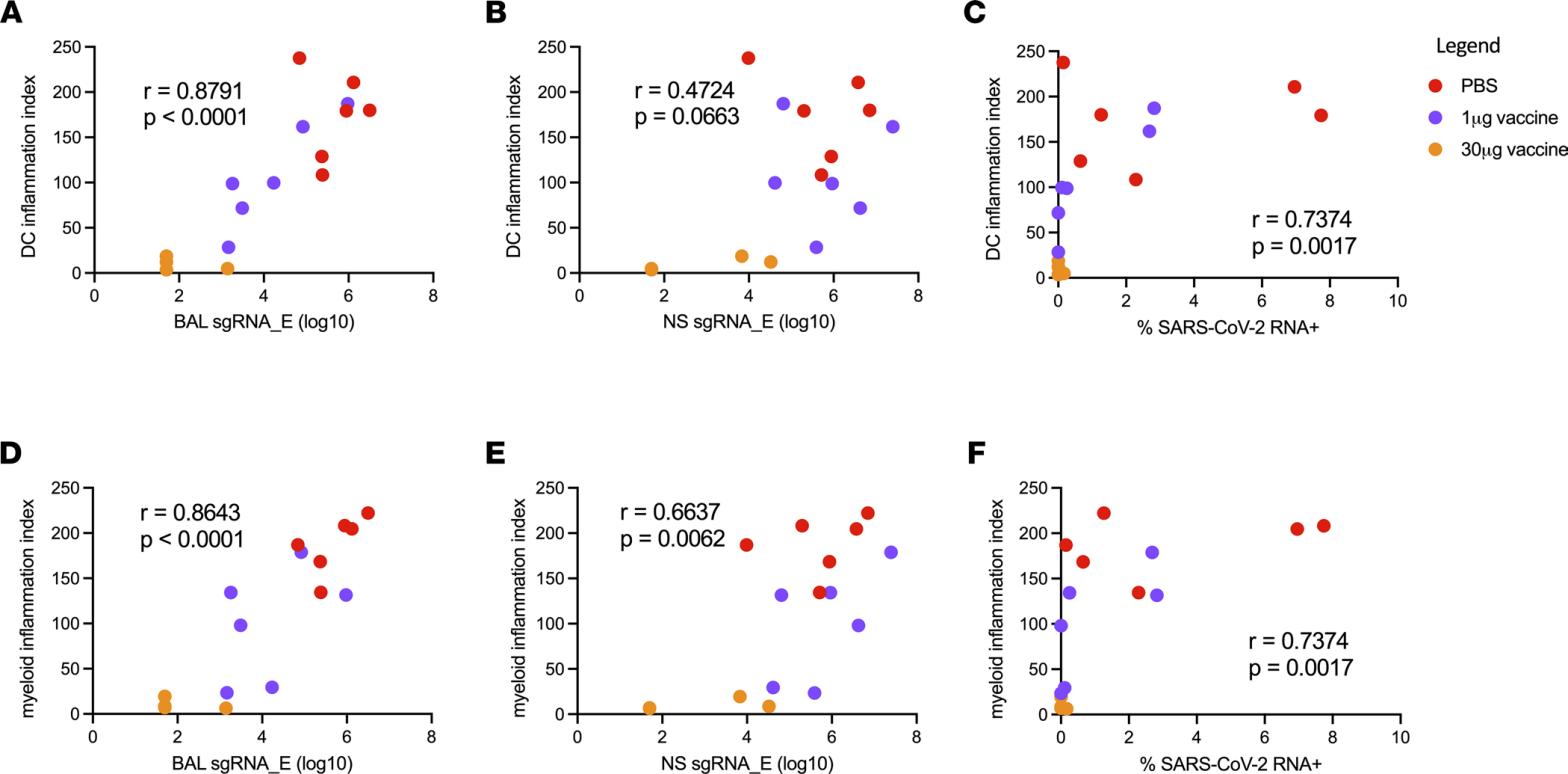
Viral load versus inflammation 2 days after infection. (**A**) DC inflammation index score versus BALF sgRNA (E gene). (**B**) DC inflammation index score versus nasal swab sgRNA (E gene). (**C**) DC inflammation index score versus SARS-CoV-2 RNA^+^ cell fraction. (**D**) Macrophage inflammation index score versus BALF sgRNA (E gene). (**E**) Macrophage inflammation index score versus nasal swab sgRNA (E gene). (**F**) Macrophage inflammation index score versus SARS-CoV-2 RNA^+^ cell fraction. Spearman’s correlation was used to assess significance.

To examine the relationship between the observed dose-dependent reduction in SARS-CoV-2 viral burden and inflammation in the BALF of mRNA-1273–vaccinated animals after SARS-CoV-2 challenge, we compared viral RNA measures to cellular inflammatory responses. Strikingly, cell-free SARS-CoV-2 RNA load positively correlated with the previously defined inflammation index score of both BALF dendritic and myeloid cell compartments on day 2 after infection across all study groups (Figure 4, A and D, and Supplemental Figure 5). However, nasal swab viral RNA load poorly correlated with dendritic cell inflammation and correlated only weakly with myeloid inflammation (Figure 4, B and E, and Supplemental Figure 5). Cell-associated viral RNA loads in the BALF also correlated with the inflammation score for both dendritic and myeloid compartments (Figure 4, C and F). These results demonstrate that viral burden in the BALF, but not nasal environment, correlates with the amount of lower respiratory tract inflammation following SARS-CoV-2 challenge.

### Prechallenge immune profiles predict lung inflammation following SARS-CoV-2 challenge.

**Figure 5:**
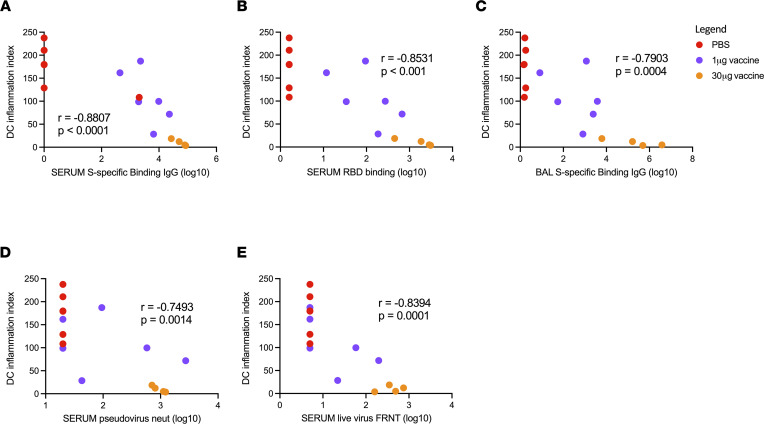
Relationship between antibody titers and SARS-CoV-2–elicited inflammation. (**A**) Relationship between prechallenge serum S-specific IgG titers (week 8 after vaccination) and DC inflammation on day 2 after challenge. (**B**) Relationship between prechallenge serum RBD-specific IgG titers (week 8 after vaccination) and DC inflammation on day 2 after challenge. (**C**) Relationship between prechallenge BALF S-specific IgG titers (week 6 after vaccination) and DC inflammation on day 2 after challenge. (**D**) Relationship between prechallenge serum pseudovirus neut titers (week 8 after vaccination) and DC inflammation on day 2 after challenge. (**E**) Relationship between prechallenge serum live virus focus reduction neutralization test (FRNT) (week 8 after vaccination) and DC inflammation on day 2 after challenge. Spearman’s correlation was used to assess significance.

SARS-CoV-2–specific antibody titers have been implicated in mRNA vaccination–mediated protection from SARS-CoV-2 infection in both humans and NHPs (30, 31, 33), but the relationship between specific antibody titers and lower respiratory tract inflammation is not clear. To this end, we incorporated previously published data (30) on serum levels of full-length spike protein and receptor-binding domain–specific (RBD-specific) titer IgG present immediately before SARS-CoV-2 challenge in these animals into our analysis. Prechallenge (8 weeks after initial vaccine dose) serum titers of both spike- and RBD-specific IgG were negatively correlated with dendritic cell inflammation scores in the lower respiratory tract 2 days after SARS-CoV-2 challenge across all study groups (Figure 5, A and B). This relationship was also observed with prechallenge spike-specific IgG titers in the BALF (Figure 5C). Furthermore, serum-neutralizing antibody responses assessed by both pseudovirus and live-virus neutralization assays were also associated with reduced DC inflammatory responses (Figure 5, D and E). In addition, prechallenge antibody titers were also observed to strongly correlate with myeloid cell inflammation scores (Supplemental Figure 6). These data suggest that SARS-CoV-2–specific antibody titers in mRNA-1273–vaccinated macaques function as a powerful predictor of SARS-CoV-2–elicited inflammation in the lower respiratory tract.

### Effect of mRNA-1273 vaccination on inflammation elicited by SARS-CoV-2 B.1.351/Beta variant.

**Figure 6:**
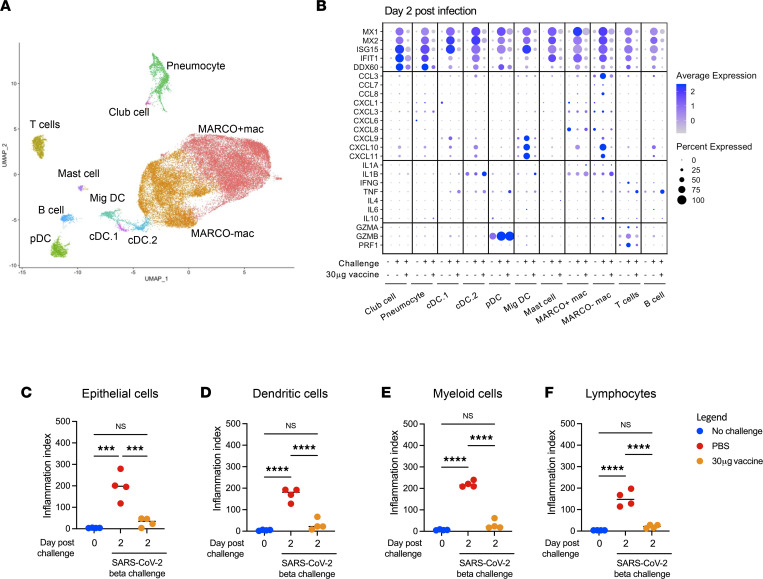
Transcriptional signatures of SARS-CoV-2 B.1.351/Beta–elicited inflammation. (**A**) UMAP projection of BALF cells from SARS-CoV-2 B.1.351/Beta challenge. (**B**) Expression of inflammatory markers and cytokines/chemokines in all annotated cells 2 days after SARS-CoV-2 B.1.351/Beta infection. (**C**) Inflammatory index scores in epithelial cells. (**D**) Inflammatory index scores in dendritic cells. (**E**) Inflammatory index scores in myeloid cells. (**F**) Inflammatory index scores in lymphocytes. ***P < 0.001, ****P < 0.0001, 1-way ANOVA with correction for multiple comparisons.

Having established the lower respiratory tract profile associated with SARS-CoV-2 USA-WA1/2020 infection — and how prior mRNA-1273 immunization blunts infection-attendant inflammation in this macaque model — we sought to expand and validate our observations in an independent experiment using a SARS-CoV-2 variant challenge. We again utilized scRNA-Seq to analyze BALF-resident cells isolated on day 2 after challenge with the SARS-CoV-2 B.1.351/Beta variant in naive animals or animals vaccinated twice with 30 μg mRNA-1273. The same populations of BALF-resident cells identified after WA-1 challenge were observed following B.1.351/Beta challenge (Figure 6A and Supplemental Figure 7). However, unlike USA-WA1/2020 challenge, infection with B.1.351/Beta resulted in a significant perturbation in the abundance of multiple cell types, including pDCs and migratory DCs (Supplemental Figure 8). These changes in cellularity were not observed in mRNA-1273–vaccinated animals, and the production of chemokines, cytokines, and cytolytic factors were again suppressed in vaccinated animals relative to their unvaccinated counterparts (Figure 6B). Vaccination with mRNA-1273 also suppressed SARS-CoV-2–associated inflammation observed in epithelial cells (Figure 6C), dendritic cells (Figure 6D), myeloid cells (Figure 6E), and lymphocytes (Figure 6F). The frequency of SARS-CoV-2 RNA^+^ cells in BALF was also reduced by mRNA-1273 vaccination, with the greatest number of viral RNA^+^ cells again found in the MARCO^–^ macrophage cluster (Supplemental Figure 9). In their totality, these results indicate that vaccination with mRNA-1273 is capable of limiting lower airway inflammation in macaques following challenge with multiple antigenically and evolutionary divergent strains of SARS-CoV-2.

## Discussion

In this study, we sought to provide functional and mechanistic insight into the properties of mRNA-1273–elicited protection from SARS-CoV-2 challenge in a widely used NHP model of mild-to-moderate COVID-19 disease. While immune correlates of protection from symptomatic SARS-CoV-2 infection are currently being assessed and defined in both clinical and preclinical studies, information is more limited on the effect of vaccine-elicited immunity on SARS-CoV-2–induced inflammation in the lungs at the single-cell level. Here, we utilized scRNA-Seq technology to analyze BALF cells from mRNA-1273–vaccinated animals that were subsequently challenged with SARS-CoV-2 to define the transcriptional signatures of infection and to ascertain how this inflammatory response is modulated by vaccine-elicited adaptive immune responses in a dose-dependent fashion. SARS-CoV-2 infection induced a robust inflammatory response in all unvaccinated animals that was suppressed in a dose-dependent fashion by mRNA-1273 vaccination. Notably, migratory DCs and MARCO^–^ macrophages appeared to be the most responsive cell types in the lower respiratory tract to SARS-CoV-2 infection, as indicated by chemokine and cytokine production. Cell-associated SARS-CoV-2 viral RNA was readily detected in the BALF of unvaccinated animals, restricted by mRNA-1273 vaccination, and correlated with dendritic and myeloid cell inflammation.

It was previously established that S-specific antibody responses elicited by mRNA-1273 vaccination correlate with upper and lower airway control of SARS-CoV-2 replication in macaques after challenge (30). Here, we further demonstrate, in these same animals, that the prechallenge antibody profile, including titers of binding and neutralizing antibody, predicted and inversely correlated with the inflammatory profile within the lung across multiple cell types. The high degree of correlation between prechallenge antibody titers, postchallenge viral loads, and postchallenge inflammation suggests a model of mRNA-1273–mediated protection from SARS-CoV-2 challenge in NHPs. Namely, neutralizing antibody levels determine the burden of viral replication, and the amount of virus persisting in the upper respiratory tract drives secondary viral dissemination to the lower respiratory tract and infection-attendant inflammation. The absence of inflammation and viral RNA in the lower respiratory tract of SARS-CoV-2–challenged animals just 2 days after infection supports the high level of efficacy of mRNA vaccination against lower respiratory infection and pathology.

Examination of human BALF from patients with mild and severe COVID-19 has been used to distinguish an inflammatory signature associated with severity. This signature includes expression of genes for chemokine production, proinflammatory cytokines, and activated phenotypic markers within resident and infiltrating cells (5–10). Several studies have described the potential role of neutrophils and NETosis in local lung pathology as well as shifts in monocyte and macrophage populations toward an inflammatory phenotype (5–7, 9, 10). Reinforcing the critical role of type I IFN in the antiviral response, many studies have also identified strong type I IFN signatures in single immune cells from patients with COVID-19, although the relationship between this cytokine profile and disease severity is still uncertain (12, 34). The type I IFN signature identified in our analysis of SARS-CoV-2–challenged rhesus macaques mirrors many of the features observed in patients experiencing acute SARS-CoV-2 infection, including broadly elevated expression of gene products, such as *MX1*, *ISG15*, and the macrophage/DC-restricted production of chemokines such as *CXCL10* (5–10). However, the transient nature of SARS-CoV-2 infection in rhesus macaques means that some features of SARS-CoV-2 infection observed in humans — such as the recruitment and activation of dysregulated cytotoxic T cells in severe COVID-19 — are not reflected in our analysis.

Our observation of stronger correlation between BALF inflammatory immune cell gene signatures and BALF viral burden than that of nasopharyngeal swabs suggests that inflammation-driven lung pathology is directly influenced by local viral replication. However, given the migratory nature of these cell populations and the relatively low abundance of viral RNA in the lower respiratory tract, the possibility that these cells were stimulated by viral ligands at other anatomical sites cannot be discounted. Furthermore, the cells that were found to be positive for SARS-CoV-2 RNA represented a range of cell types. Although the range of cell types expressing ACE2 and, therefore, permissive to viral entry is wide (35), these populations may not represent bona fide productively infected cells. Rather, cells may acquire viral RNA through phagocytic mechanisms, for example. Indeed, the macrophage population identified in our analysis as harboring the most SARS-CoV-2 RNA is defined by the expression of the scavenger receptor molecule MARCO, which may facilitate the clearance and uptake of apoptotic or necrotic SARS-CoV-2–infected cells that are not otherwise effectively captured in our scRNA-Seq analysis. Despite these potential caveats, our findings were validated by the challenge of animals with the SARS-CoV-2 B.1.351/Beta variant, wherein mRNA-1273 vaccination also prevented infection-induced lung inflammation.

There are some limitations of this study to consider. First, the relatively transient and self-limiting nature of SARS-CoV-2 infection and infection-elicited inflammation in macaques makes it difficult to place these observations into context of human disease. Many of the pathways, cell types, transcriptional signatures, and correlates of protection identified in our analysis have also been defined in humans with acute COVID-19, but the magnitude and timing of the events may not be homologous. Second, the route of virus administration has been shown to influence infection and inflammation in other models of SARS-CoV-2 challenge (36), so that the i.n./i.t. route of infection used in this study may result in subtly different features of infection and inflammation than aerosol-mediated infection. Finally, our study exclusively focuses on the relationship between prechallenge antibody titers and SARS-CoV-2–elicited inflammation, leaving unaddressed the potential contribution of spike-reactive memory T cells in the model. However, the kinetics of SARS-CoV-2 replication in rhesus macaques means that memory T cells are unlikely to have an opportunity to expand and traffic to the lung within the window of active viral replication. Even in naive animals, viral control is achieved prior to the development of tissue-specific SARS-CoV-2 T cell responses, and several studies have now shown that depletion of T cells in the macaque model only modestly affects viral control, especially in the setting of high levels of neutralizing antibodies (37, 38).

In conclusion, this study defines the lower respiratory tract cellular and transcriptional signature associated with SARS-CoV-2 infection in macaques using 2 distinct viral variants and identifies conserved signatures of vaccine-elicited protection from infection-attendant inflammation. These data emphasize the contribution of inflammatory/migratory DCs and macrophages to lower respiratory tract inflammation following SARS-CoV-2 infection and define a critical relationship among antibody titers, postchallenge viral burden, and broad infection-elicited inflammation.

## Methods

### Vaccine formulation.

mRNA encoding a sequence-optimized and prefusion-stabilized SARS-CoV-2 S-2P protein (39, 40) was synthesized in vitro and formulated as previously reported (19, 41, 42).

### Rhesus macaque vaccination model.

Three- to eight-year-old rhesus macaques of Indian origin were sorted by sex, age, and weight and then stratified into groups as previously described (30, 31). Animals were immunized intramuscularly at week 0 and at week 4 with 1 μg or 30 μg mRNA-1273 in 1 mL PBS into the right hind leg. Placebo-control animals were administered an equal volume of PBS. Studies were conducted at Bioqual Inc. (Rockville, Maryland, USA). Postvaccination antibody titers were generated as previously described (30, 41, 43–46) and previously reported by Corbett et al. (30).

### USA-WA1/2020 challenge.

At week 8 after initial vaccination (4 weeks after boost), all animals were challenged with a total dose of 8 × 10^5^ PFU SARS-CoV-2 as previously described (30). The stock of 1.99 × 10^6^ TCID_50_ or 3 × 10^6^ PFU/mL SARS-CoV-2 USA-WA1/2020 strain (BEI NR-70038893) was diluted and administered in 3 mL doses by the i.t. route and in 1 mL doses by the i.n. route (0.5 mL per nostril). Postchallenge SARS-CoV-2 sgRNA burden in nasal swabs and BAL were determined as previously described (19, 30) and previously reported by Corbett et al. (30).

### B.1.351 challenge.

At week 8 after initial vaccination (4 weeks after boost), NHPs were challenged with a total dose of 5 × 10^5^ PFU SARS-CoV-2 B.1.351 strain as previously described (31). The viral inoculum was administered as 3.75 × 10^5^ PFU in 3 mL i.t. and 1.25 × 10^5^ PFU in 1 mL i.n. in a volume of 0.5 mL into each nostril.

### scRNA-Seq library generation.

Freshly isolated BALF suspensions were prepared for scRNA-Seq using the Chromium Single-Cell 5′ Reagent v2 kit or NextGEM v1.0 kit and the Chromium Single-Cell Controller (all from 10x Genomics) (47). 2000 to 8000 cells per reaction suspended at a density of 50–500 cells/μL in PBS plus 0.5% FBS were loaded for gel bead-in-emulsion generation and barcoding. Reverse transcription, reverse transcription cleanup, and cDNA amplification were performed to isolate and amplify cDNA for downstream library construction according to the manufacturer’s protocol (10x Genomics). Libraries were constructed using the Chromium Single-Cell 5′ reagent kit and i7 Multiplex Kit (10x Genomics) according to the manufacturer’s protocol.

### Sequencing.

scRNA-Seq 5′ gene expression libraries were sequenced on an Illumina NovaSeq 6000 instrument using the S1, S2, or S4 reagent kits (300 cycles). Libraries were balanced to allow for approximately 150,000 reads/cell for 5′ gene expression libraries. Sequencing parameters were set for 150 cycles for Read1, 8 cycles for Index1, and 150 cycles for Read2. Prior to sequencing, library quality and concentration were assessed using an Agilent 4200 TapeStation with High Sensitivity D5000 ScreenTape Assay and Qubit Fluorometer (Thermo Fisher Scientific) with dsDNA BR assay kit according to the manufacturer’s recommendations.

### scRNA-Seq gene expression analysis/visualization.

5′ gene expression alignment from all BALF samples was performed using the 10x Genomics Cell Ranger pipeline (47). Sample demultiplexing, alignment, barcode/UMI filtering, and duplicate compression were performed using the Cell Ranger software package (10x Genomics, v2.1.0) and bcl2fastq2 (Illumina, v2.20) according to the manufacturers’ recommendations, using the default settings and mkfastq/count commands, respectively. All reads were trimmed to 26 × 98 bp for gene expression analysis. Transcript alignment was performed against a *Macaca mulataa* reference library generated using the Cell Ranger mkref command, the Ensembl Mmul_10 top-level genome FASTA, and the corresponding Ensembl v100 gene GTF.

Multisample integration, data normalization, dimensional reduction, visualization, and differential gene expression were performed using the R package Seurat (v4.0.0) (48, 49). All data sets were filtered to only contain cells with between 200 and 5000 unique features and more than 12.5% mitochondrial RNA gene content (defined as expression of the following mitochondrial gene products: *ND1*, *ND2*, *COX1*, *COX2*, *ATP8*, *ATP6*, *COX3*, *ND3*, *ND4L*, *ND4*, *ND5*, and *CYTB*). To eliminate erythrocyte contamination, data sets were additionally filtered to contain cells with less than a 10% erythrocytic gene signature (defined as HBA and HBB). Data were scaled, normalized, and transformed prior to multisample integration using the negative binomial regression model of the Seurat SCTransform() function, additionally regressing-out the contribution of imputed cell cycle to the normalized data set (50). SelectIntegrationFeatures() and PrepSCTIntegration() functions were used to identify conserved features for data set integration, and final data set anchoring/integration was performed using FindIntegrationAnchors() and IntegrateData() functions, with the day 2 30 μg vaccine samples used as reference data sets. Principal component analysis (PCA) was performed using variable genes defined by SCTransform().

For the USA-WA1/2020 data set, the first 40 resultant principal components (PCs) were initially used to perform a UMAP dimensional reduction of the data set (RunUMAP()) and to construct a shared nearest neighbor graph [SNN; FindNeighbors()]. This SNN was used to cluster the data set [FindClusters()] with default parameters and resolution set to 0.7. From this initial clustering, a population of low-viability cells was identified and removed from the analysis, after which the data set PCA was rerun, and the first 35 resultant PCs were used to perform a UMAP dimensional reduction of the data set [RunUMAP()] and to construct a SNN [FindNeighbors())]. This SNN was used to cluster the data set [FindClusters()] with default parameters and resolution set to 1.5. The resultant clusters were assigned to the following cell types based on the expression of the indicated gene products: club cell (*SCGB1A1*, *SCGB3A1*), pneumocyte (*PIFO*, *SNTN*, *FOXJ1*), MARCO^–^ macrophage (*MRC1*, *APOE*), MARCO^+^ macrophage (*MRC1*, *APOE*, *MARCO*), cDC.1 (*CLEC9A*, *XCR1*), cDC.2 (*CD1C, CLEC10A*), pDC (*GZMB, IRF7*), MigDCs (*CCR7*, *BIRC3*), Mast cell (*CPA3, GATA2*), CD4^+^ T cell (*CD3E, CD40LG*), CD8^+^ T cell (*CD3E*, *CD8A*), and B cell (*CD19*, *MS4A1*).

For the B.1.351/Beta data set, the first 31 resultant PCs were initially used to perform a UMAP dimensional reduction of the data set [RunUMAP()] and to construct a SNN [FindNeighbors()]. This SNN was used to cluster the data set [FindClusters()] with default parameters and resolution set to 1.7. From this initial clustering a population of low-viability cells was identified and removed from the analysis, after which the data set PCA was rerun, and the first 31 resultant PCs were used to perform a UMAP dimensional reduction of the data set [RunUMAP()] and to construct a SNN [FindNeighbors()]. This SNN was used to cluster the data set [FindClusters()] with default parameters and resolution set to 1.7. The resultant clusters were assigned to the following cell types based on the expression of the indicated gene products: club cell (*SCGB1A1*, *SCGB3A1*), pneumocyte (*PIFO*, *SNTN FOXJ1*), MARCO^–^ mac (*MRC1*, *APOE*), MARCO^+^ mac (*MRC1*, *APOE*, *MARCO*), cDC.1 (*CLEC9A*, *XCR1*), cDC.2 (*CD1C*, *CLEC10A*), pDC (*GZMB*, *IRF7*), MigDC (*CCR7*, *BIRC3*), Mast cell (*CPA3*, *GATA2*), CD4^+^ T cell (*CD3E*, *CD40LG*), CD8^+^ T cell (*CD3E*, *CD8A*), and B cells (*CD19*, *MS4A1*).

Following data set integration and dimensional reduction/clustering, gene expression data were log transformed and scaled by a factor of 10,000 using the NormalizeData() function. These normalized gene expression data were used to determine cellular cluster identity by utilizing the Seurat application of a Wilcoxon’s rank-sum test [FindAllMarkers()] and comparing the resulting differential expression data with known cell linage–specific gene sets. Differential gene expression analysis between study time points was performed using normalized gene expression data and the Wilcoxon’s rank-sum test with implementation in the FindMarkers() function, with a log_2_ fold change threshold of 0.5 and min.pct of 0.25. Bonferroni’s correction was used to control for FDR, with a corrected *P* value of less than 0.05 considered significant. Inflammation index scores were calculated using the AverageExpression() function of Seurat to determine the summed averaged normalized/exponentiated expression of MX1, MX2, IFIT1, IFIT2, IFIT3, IFI6, ISG15, and ISG20 in the indicated cell populations. Network analysis of differentially expressed genes was performed using QIAGEN IPA v01-20-04 (51).

### Identification of SARS-CoV-2 RNA^+^ cells.

Quantification and alignment of cell-associated SARS-CoV-2 RNA from was performed using the 10x Genomics Cell Ranger pipeline(47). Sample demultiplexing, alignment, barcode/UMI filtering, and duplicate compression was performed using the Cell Ranger software package (10× Genomics, v2.1.0) and bcl2fastq2 (Illumina, v2.20) according to the manufacturers’ recommendations, using the default settings and mkfastq/count commands, respectively. The resulting untrimmed (150 × 150 bp) FASTQs were filtered to only contain reads that did not align to the *Macaca mulataa* genome using seqfilter and the read annotation from the Cellranger alignment performed on the trimmed FASTQs performed against the Ensembl Mmul_10 genome described above. Transcript alignment was performed against a SARS-CoV-2 reference generated using the Cell Ranger mkref command and the SARS-CoV-2 reference genome (strain USA-WA1/2020) FASTA and the corresponding gene GTF.

### Data and material availability.

Data tables for expression counts and unprocessed raw data from the scRNA-Seq analysis were deposited in NCBI’s Gene Expression Omnibus (GEO GSE190913 for USA-WA1/2020 challenge and GEO GSE190165 for B.1.351/Beta challenge).

### Statistics.

Differential gene expression analysis of scRNA-Seq data was performed using normalized gene expression counts and the Wilcoxon’s rank-sum test in the Seurat FindMarkers() function. A log_2_ fold change threshold for gene expression changes of 0.5 and min.pct of 0.25 was used for all comparisons, and a Bonferroni’s correction was used to control for FDR. Corrected *P* values of less than 0.05 were considered significant in conjunction with the additional filters above. Paired 2-tailed t tests, 1-way ANOVAs, and Spearman’s correlation tests were performed using GraphPad Prism 9 Software. *P* values of less than 0.05 were considered significant.

### Study approval.

Animal experiments were performed in compliance with all pertinent NIH regulations and with approval from the Animal Care and Use Committees of the Vaccine Research Center and Bioqual Inc. Research was conducted under an approved animal use protocol in an AAALAC-accredited facility in compliance with the Animal Welfare Act and other federal statutes and regulations relating to animals and experiments involving animals; it adheres to principles stated in the *Guide for the Care and Use of Laboratory Animals* (National Academies Press, 2011).

## Author contributions

RS, MR, and KEF designed the study. KV and TL generated the data. ATW, KN, HF, KEF, MR, DLB, JRC, and RS analyzed and interpreted the data. ATW, KN, and JRC wrote the paper with assistance from all coauthors.

## Supplementary Material

Supplemental data

Supplemental data set 1

Supplemental data set 2

## Version 1 06/02/2022

In-Press Preview

## Version 2 07/08/2022

Electronic publication

## References

[1] Dong E (2020). An interactive web-based dashboard to track COVID-19 in real time. Lancet Infect Dis.

[2] Richardson S (2020). Presenting characteristics, comorbidities, and outcomes among 5700 patients hospitalized with COVID-19 in the New York City area. JAMA.

[3] Gupta S (2020). Factors associated with death in critically ill patients with Coronavirus Disease 2019 in the US. JAMA Intern Med.

[4] Chua RL (2020). COVID-19 severity correlates with airway epithelium-immune cell interactions identified by single-cell analysis. Nat Biotechnol.

[5] Grant RA (2021). Circuits between infected macrophages and T cells in SARS-CoV-2 pneumonia. Nature.

[6] Schulte-Schrepping J (2020). Severe COVID-19 is marked by a dysregulated myeloid cell compartment. Cell.

[7] Wilk AJ (2020). A single-cell atlas of the peripheral immune response in patients with severe COVID-19. Nat Med.

[8] Wen W (2020). Immune cell profiling of COVID-19 patients in the recovery stage by single-cell sequencing. Cell Discov.

[9] Silvin A (2020). Elevated calprotectin and abnormal myeloid cell subsets discriminate severe from mild COVID-19. Cell.

[10] Chen H (2021). SARS-CoV-2 activates lung epithelial cell proinflammatory signaling and leads to immune dysregulation in COVID-19 patients. EBioMedicine.

[11] Yao C (2021). Cell-type-specific immune dysregulation in severely ill COVID-19 patients. Cell Rep.

[12] Lee JS (2020). Immunophenotyping of COVID-19 and influenza highlights the role of type I interferons in development of severe COVID-19. Sci Immunol.

[13] Wong LYR, Perlman S (2022). Immune dysregulation and immunopathology induced by SARS-CoV-2 and related coronaviruses - are we our own worst enemy?. Nat Rev Immunol.

[14] Speranza E (2021). Single-cell RNA sequencing reveals SARS-CoV-2 infection dynamics in lungs of African green monkeys. Sci Transl Med.

[15] Hoang TN (2021). Baricitinib treatment resolves lower-airway macrophage inflammation and neutrophil recruitment in SARS-CoV-2-infected rhesus macaques. Cell.

[16] Klasse PJ (2021). Immunogenicity of clinically relevant SARS-CoV-2 vaccines in nonhuman primates and humans. Sci Adv.

[17] Yu J (2020). DNA vaccine protection against SARS-CoV-2 in rhesus macaques. Science.

[18] Mercado NB (2020). Single-shot Ad26 vaccine protects against SARS-CoV-2 in rhesus macaques. Nature.

[19] Corbett KS (2020). Evaluation of the mRNA-1273 Vaccine against SARS-CoV-2 in Nonhuman Primates. N Engl J Med.

[20] Munster VJ (2020). Respiratory disease in rhesus macaques inoculated with SARS-CoV-2. Nature.

[21] King HAD (2021). Efficacy and breadth of adjuvanted SARS-CoV-2 receptor-binding domain nanoparticle vaccine in macaques. Proc Natl Acad Sci U S A.

[22] Joyce MG (2021). A SARS-CoV-2 ferritin nanoparticle vaccine elicits protective immune responses in nonhuman primates. Sci Transl Med.

[23] Lee JS (2021). Single-cell transcriptome of bronchoalveolar lavage fluid reveals sequential change of macrophages during SARS-CoV-2 infection in ferrets. Nat Commun.

[24] Tregoning JS (2021). Progress of the COVID-19 vaccine effort: viruses, vaccines and variants versus efficacy, effectiveness and escape. Nat Rev Immunol.

[25] Polack FP (2020). Safety and efficacy of the BNT162b2 mRNA Covid-19 vaccine. N Engl J Med.

[26] Baden LR (2021). Efficacy and safety of the mRNA-1273 SARS-CoV-2 vaccine. N Engl J Med.

[27] Goldberg Y (2021). Waning immunity after the BNT162b2 vaccine in Israel. N Engl J Med.

[28] Levin EG (2021). Waning immune humoral response to BNT162b2 Covid-19 vaccine over 6 months. N Engl J Med.

[29] Choi A (2021). Safety and immunogenicity of SARS-CoV-2 variant mRNA vaccine boosters in healthy adults: an interim analysis. Nat Med.

[30] Corbett KS (2021). Immune correlates of protection by mRNA-1273 vaccine against SARS-CoV-2 in nonhuman primates. Science.

[32] Madissoon E (2019). scRNA-seq assessment of the human lung, spleen, and esophagus tissue stability after cold preservation. Genome Biol.

[33] Feng S (2021). Correlates of protection against symptomatic and asymptomatic SARS-CoV-2 infection. Nat Med.

[34] Bost P (2020). Host-viral infection maps reveal signatures of severe COVID-19 patients. Cell.

[35] Lukassen S (2020). SARS-CoV-2 receptor ACE2 and TMPRSS2 are primarily expressed in bronchial transient secretory cells. EMBO J.

[37] Nelson CE Mild SARS-CoV-2 infection in rhesus macaques is associated with viral control prior to antigen-specific T cell responses in tissues. Sci Immunol.

[38] McMahan K (2021). Correlates of protection against SARS-CoV-2 in rhesus macaques. Nature.

[39] Pallesen J (2017). Immunogenicity and structures of a rationally designed prefusion MERS-CoV spike antigen. Proc Natl Acad Sci U S A.

[40] Wrapp D (2020). Cryo-EM structure of the 2019-nCoV spike in the prefusion conformation. Science.

[41] Corbett KS (2020). SARS-CoV-2 mRNA vaccine design enabled by prototype pathogen preparedness. Nature.

[42] Hassett KJ (2019). Optimization of lipid nanoparticles for intramuscular administration of mRNA vaccines. Mol Ther Nucleic Acids.

[44] Jackson LA (2020). An mRNA Vaccine against SARS-CoV-2 - Preliminary Report. N Engl J Med.

[45] Vanderheiden A (2020). Development of a rapid focus reduction neutralization test assay for measuring SARS-CoV-2 neutralizing antibodies. Curr Protoc Immunol.

[46] Katzelnick LC (2018). Viridot: An automated virus plaque (immunofocus) counter for the measurement of serological neutralizing responses with application to dengue virus. PLoS Negl Trop Dis.

[47] Zheng GX (2017). Massively parallel digital transcriptional profiling of single cells. Nat Commun.

[48] Stuart T (2019). Comprehensive integration of single-cell data. Cell.

[49] Butler A (2018). Integrating single-cell transcriptomic data across different conditions, technologies, and species. Nat Biotechnol.

[50] Hafemeister C, Satija R (2019). Normalization and variance stabilization of single-cell RNA-seq data using regularized negative binomial regression. Genome Biol.

[51] Kramer A (2014). Causal analysis approaches in Ingenuity Pathway Analysis. Bioinformatics.

