# Supplementary material for: mRNA-1273 vaccination protects against SARS-CoV-2–elicited lung inflammation in nonhuman primates
Source: JCI Insight. 2022 Jul 8;7(13):e160039. doi: 10.1172/jci.insight.160039 (PMC9310526; doi:10.1172/jci.insight.160039)
Supplement: Supplemental data [file jciinsight-7-160039-s121.pdf]

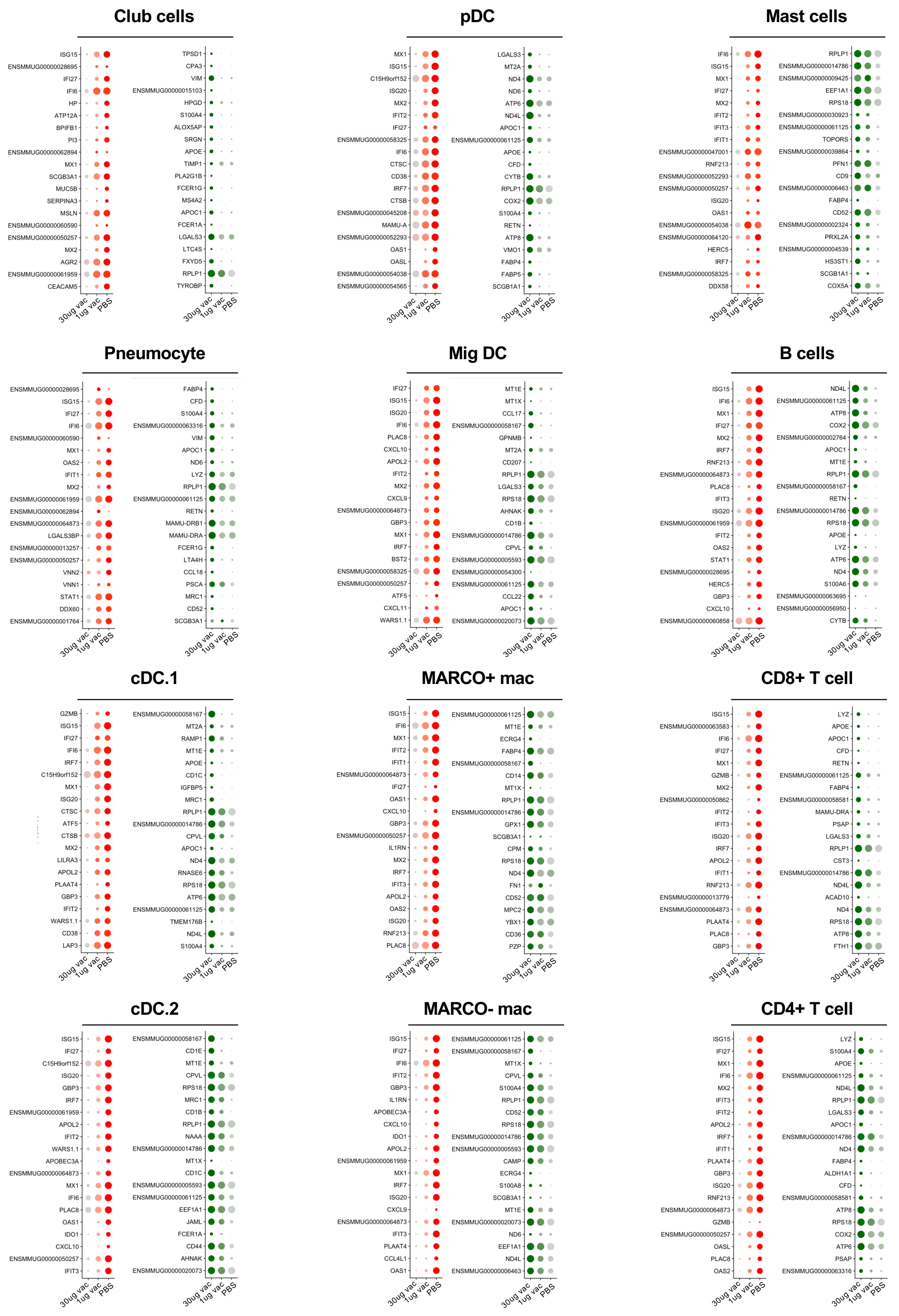


**Supplemental Figure 1: Differential gene expression analysis, day 2 post SARS-CoV-2 challenge.** The top 20 differentially expressed genes observed to be higher in unvaccinated animals than in the 30ug mRNA-1273 vaccinated animals on day 2 post challenge are shown in red. The top 20 differentially expressed genes observed to be higher in the 30ug mRNA-1273 vaccinated animals than in the unvaccinated animals on day 2 post challenge are shown in green.

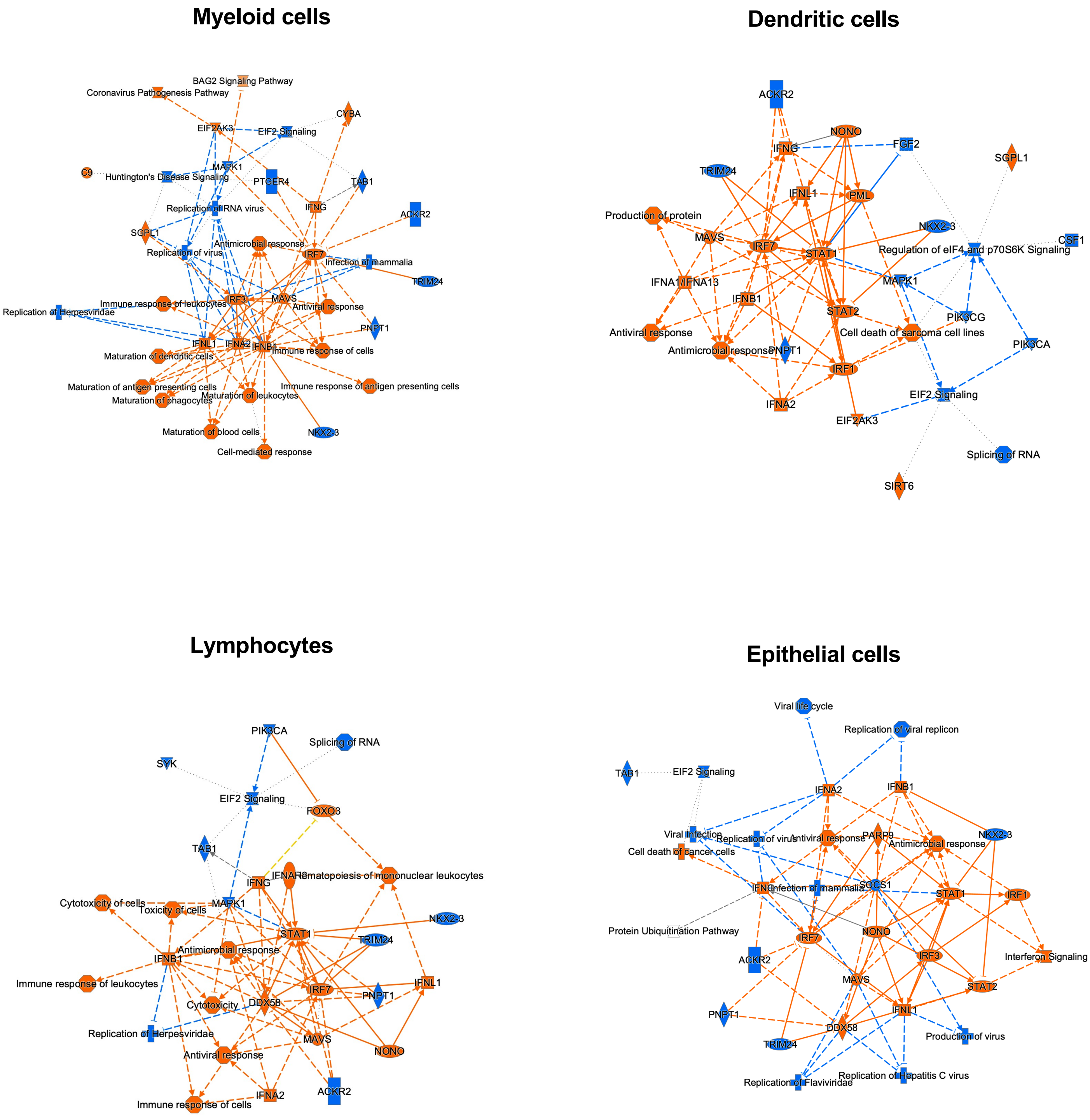


**Supplemental Figure 2: Graphical summary of cell signaling networks impacted by SARS-CoV-2 infection in unvaccinated animals (day 2 post challenge) relative to uninfected animals.** Differential gene expression analysis was performed to identified genes induced or suppressed by SARS-CoV-2 infection relative to un-infected animals. The resultant differentially expressed genes were analyzed using the Ingenuity Pathway Analysis (IPA) software package. **Orange nodes**: cell processes activated by SARS-CoV-2 infection relative to un-infected animals. **Blue nodes**: cell processes inhibited by SARS-CoV-2 infection relative to un-infected animals. **Orange lines**: interactions that lead to activation of connected nodes. **Blue line**: interactions that lead to inhibition of connected nodes. **Solid line**: direct interaction. **Dashed line**: indirect interaction.

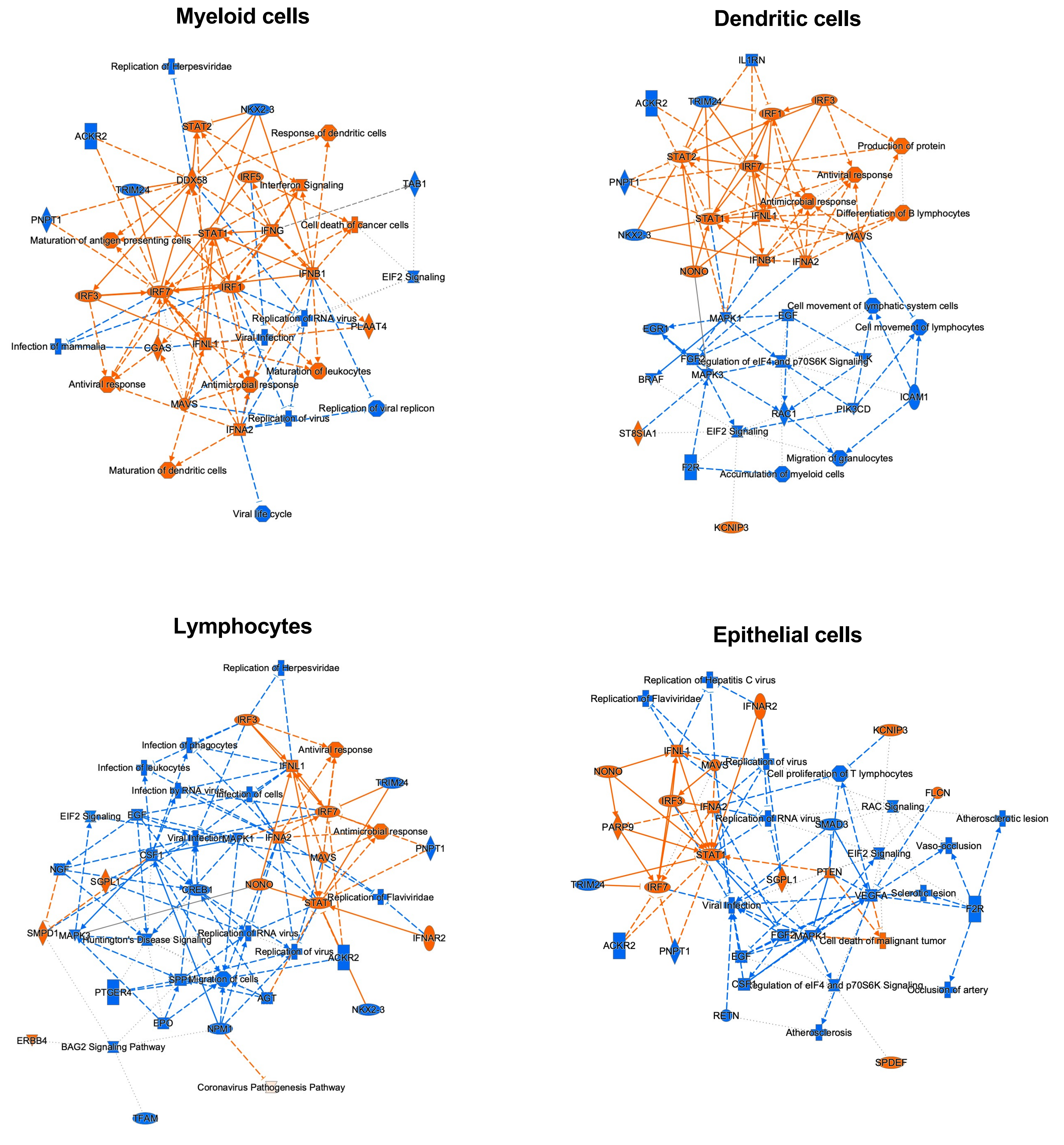


**Supplemental Figure 3: Graphical summary of cell signaling networks impacted by SARS-CoV-2 infection in <u>unvaccinated</u> animals (day 2 post challenge) relative to <u>vaccinated</u> animals (day 2 post infection).** Differential gene expression analysis was performed to identified genes induced or suppressed by SARS-CoV-2 infection relative to animals who were fully vaccinated and challenge with the same dose of SARS-CoV-2. The resultant differentially expressed genes were analyzed using the Ingenuity Pathway Analysis (IPA) software package. **Orange nodes**: cell processes activated by SARS-CoV-2 infection relative to un-infected animals. **Blue nodes**: cell processes inhibited by SARS-CoV-2 infection relative to un-infected animals. **Orange lines**: interactions that lead to activation of connected nodes. **Blue line**: interactions that lead to inhibition of connected nodes. **Solid line**: direct interaction. **Dashed line**: indirect interaction.

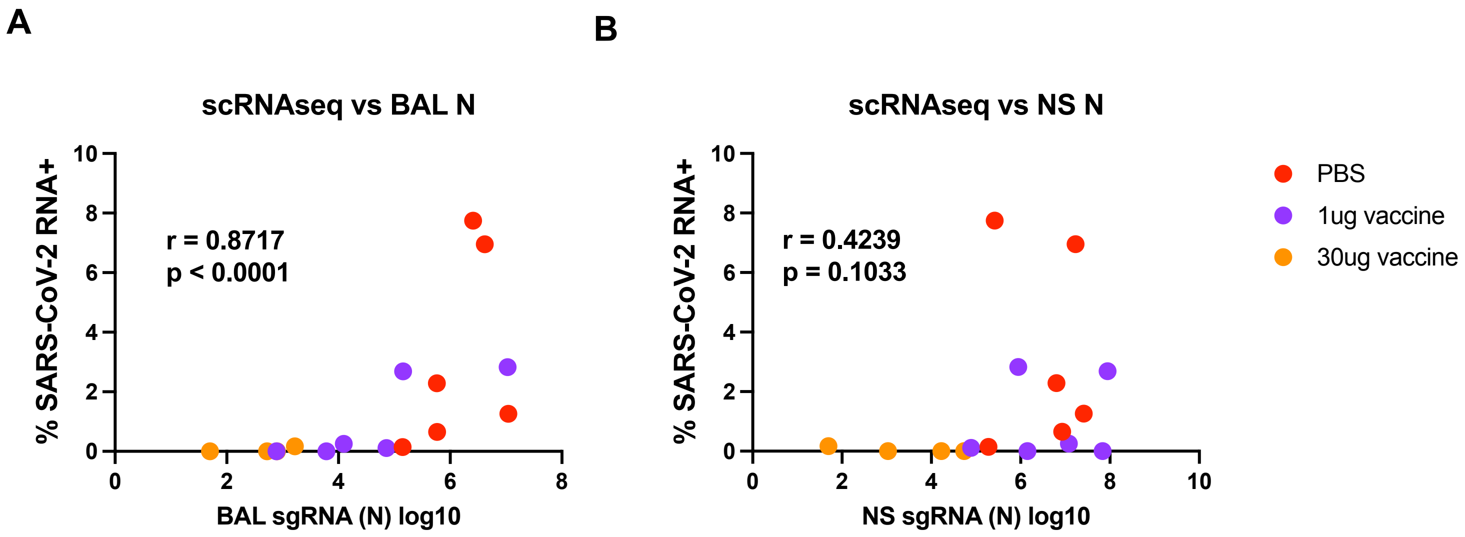


**Supplemental Figure 4:** Relationship between PCR and scRNAseq viral loads, day 2 post challenge. A) Relationship between BAL RNA load (N) and frequency of SARS-CoV-2 RNA+ cells. B) Relationship between NS RNA load (N) and frequency of SARS-CoV-2 RNA+ cells. Spearman correlation

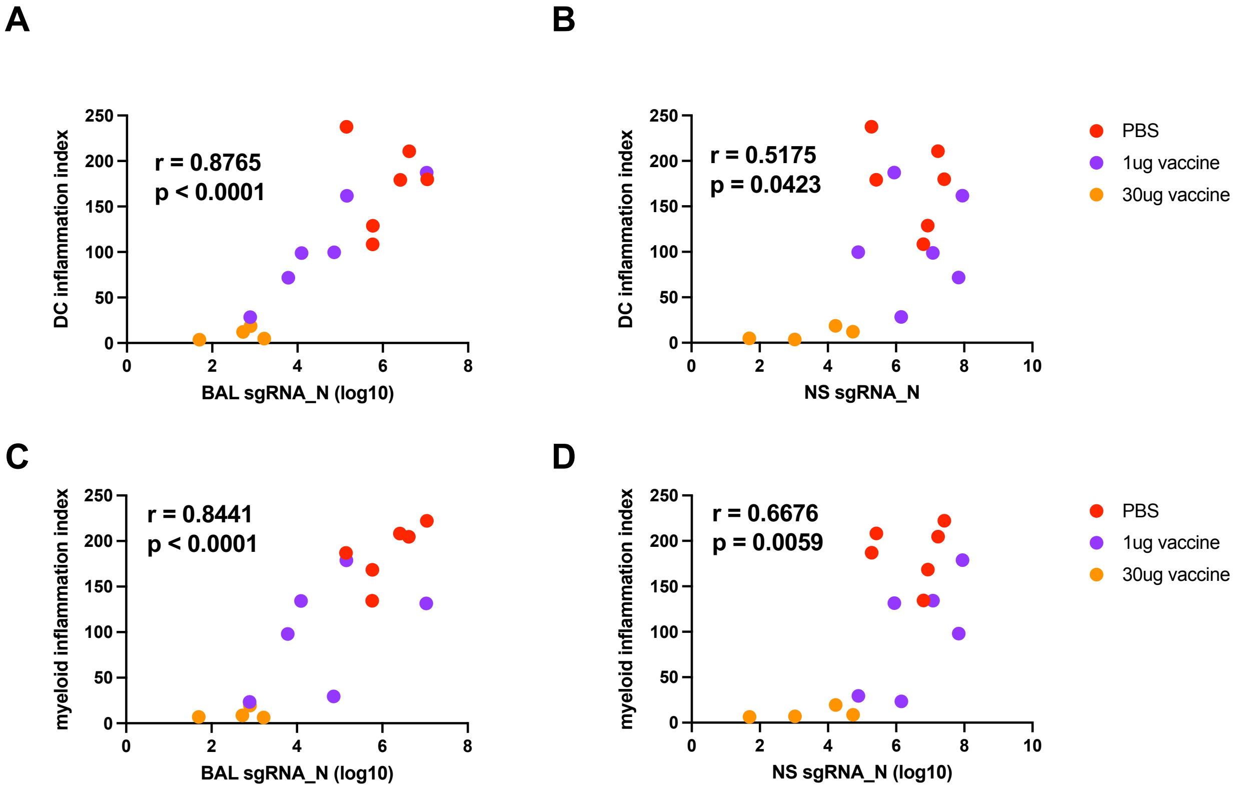


**Supplemental Figure 5: viral load vs inflammation scores, day 2 post infection**. **A)** DC Inflammation index score vs BAL sgRNA (N gene). **B)** DC Inflammation index score vs NS sgRNA (N gene). **C)** Macrophage Inflammation index score vs BAL sgRNA (N gene). **D)** Macrophage Inflammation index score vs NS sgRNA (N gene). Spearman correlation

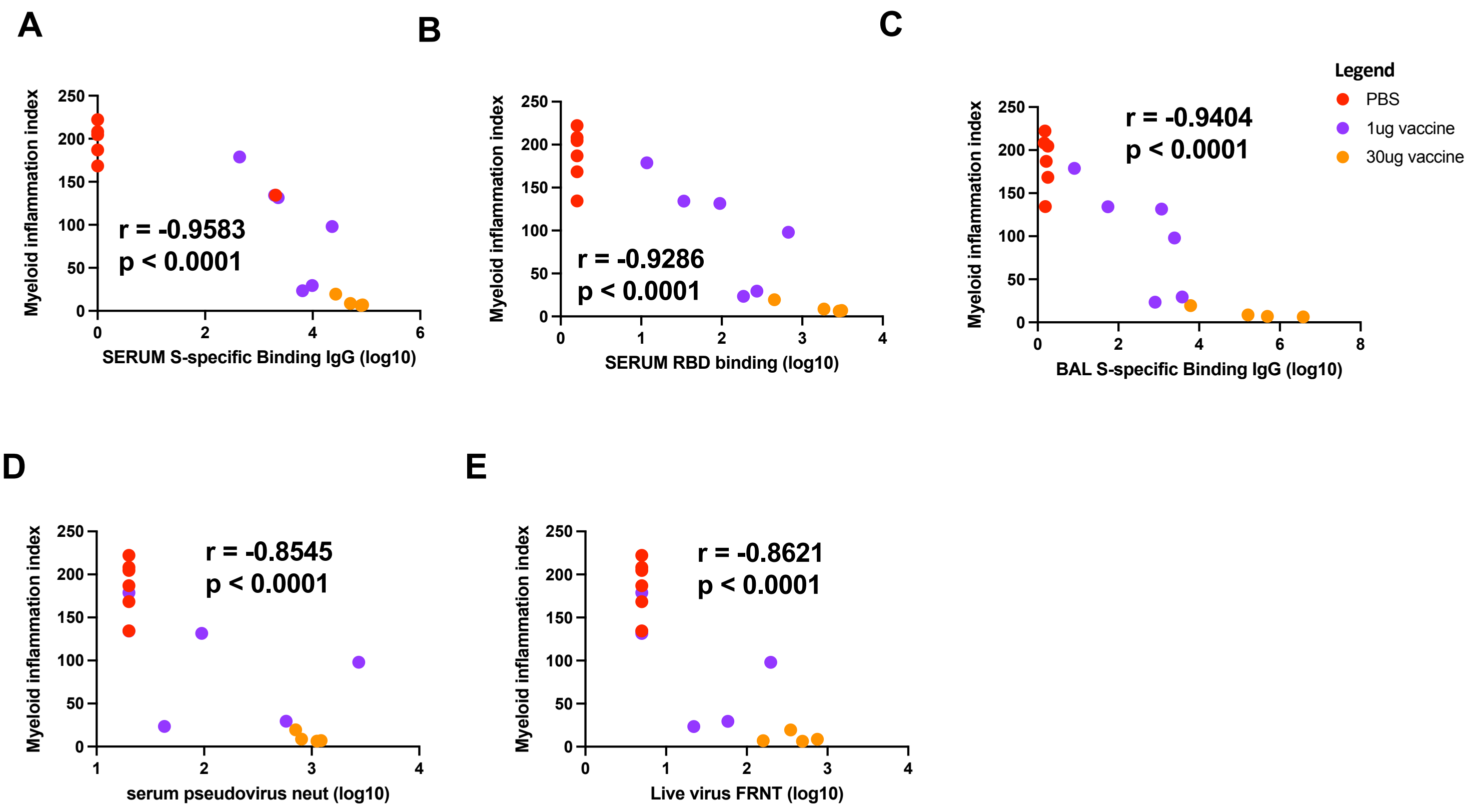


**Supplemental Figure 6. Relationship between antibody titers and SARS-CoV-2 elicited inflammation. A)** Relationship between pre-challenge serum S-specific IgG titers (wk 8 post vaccination) and myeloid inflammation on day 2 post challenge. **B)** Relationship between pre-challenge serum RBD-specific IgG titers (wk 8 post vaccination) and myeloid inflammation on day 2 post challenge. **C)** Relationship between pre-challenge BALF S-specific IgG titers (wk 6 post vaccination) and myeloid inflammation on day 2 post challenge. **D)** Relationship between pre-challenge serum pseudovirus neut titers titers (wk 8 post vaccination) and myeloid inflammation on day 2 post challenge. **E)** Relationship between pre-challenge serum live virus FRNT (wk 8 post vaccination) and myeloid inflammation on day 2 post challenge. Spearman correlation

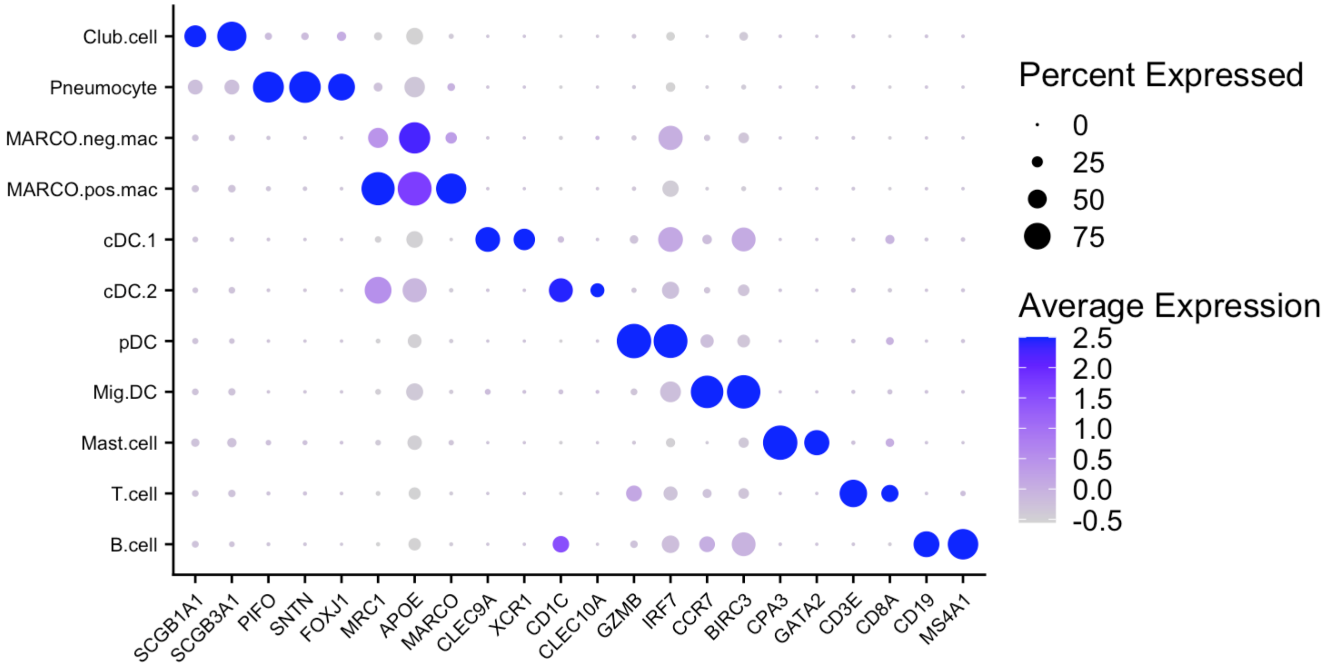


**Supplemental Figure 7: identification and quantification of BALF cells by scRNAseq.**
Expression of key linage specific genes in all annotated cell types in BALF collected post SARS-CoV-2 strain B.1.351/beta challenge

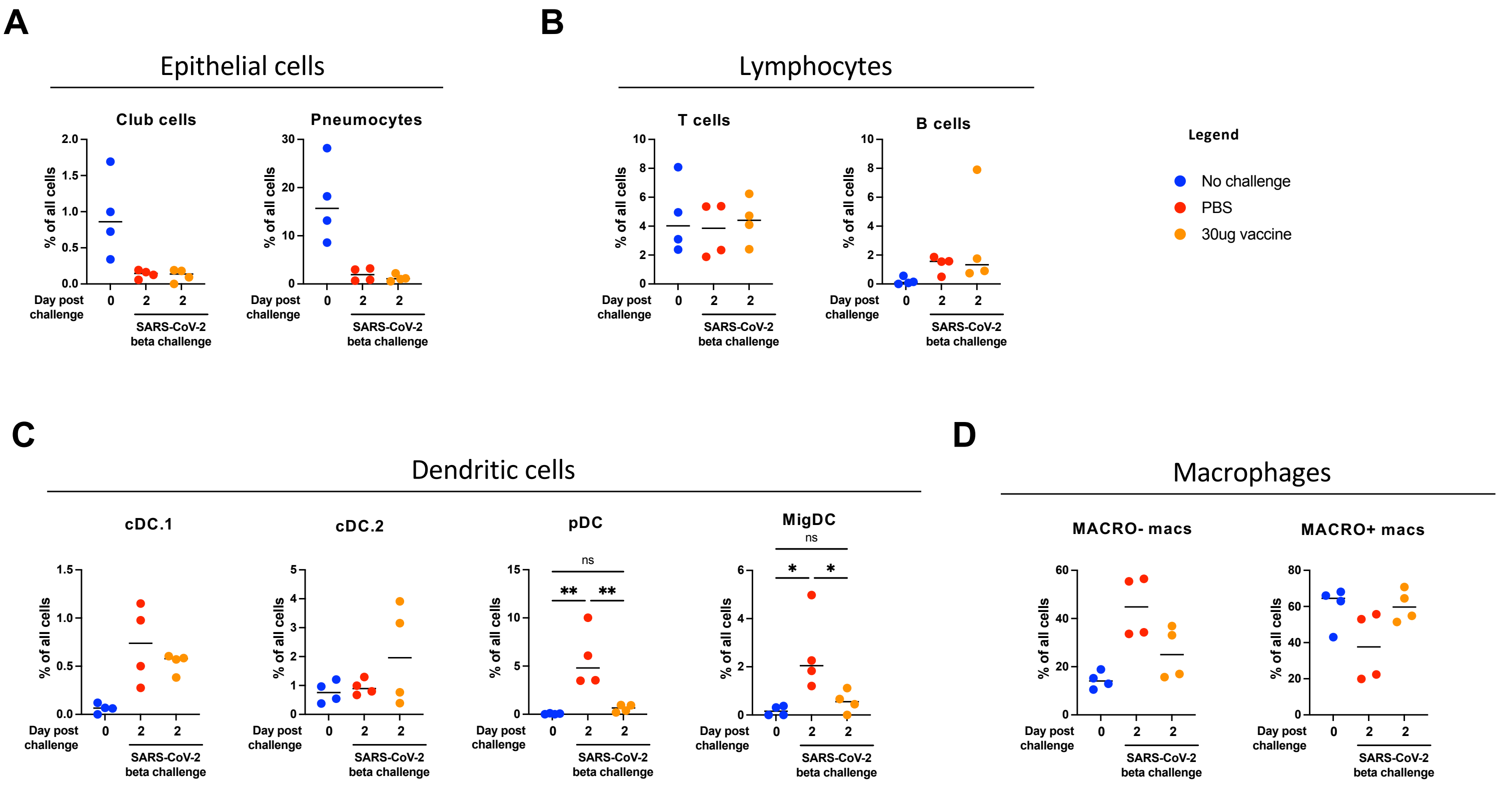


**Supplemental Figure 8: identification and quantification of BALF cells by scRNAseq following B.1.351/beta infection**. **A)** Frequency of epithelial cell populations. **B)** Frequency of lymphocyte cell populations. **C)** Frequency of dendritic cell populations. **D)** Frequency of macrophage populations. ** $p < 0.01$, * $p < 0.05$, one-way ANOVA with correction for multiple comparisons

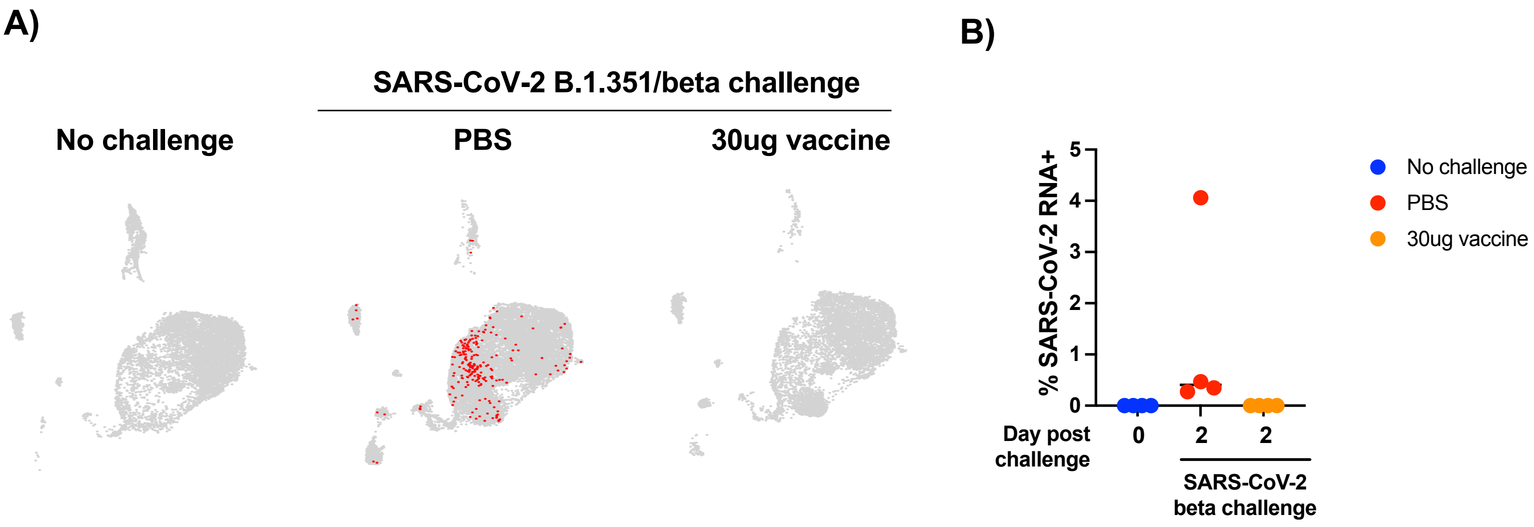


**Supplemental Figure 9: identification and quantification of SARS-CoV-2 B.1.351/beta variant RNA$^{+}$ cells. A)** Location of SARS-CoV-2 RNA$^{+}$ cells. **B)** Frequency of SARS-CoV-2 RNA$^{+}$ cell following SARS-CoV-2 B.1.351 challenge

**Supplemental Table 1: Cellular networks impacted by SARS-CoV-2 infection within myeloid cells in unvaccinated animals (day 2 post challenge) relative to un-infected animals**

| Ingenuity Canonical Pathways | -log(p-value) | Ratio | z-score |
|---|---|---|---|
| EIF2 Signaling | 5.06E+01 | 3.97E-01 | -5.461 |
| BAG2 Signaling Pathway | 2.67E+01 | 4.76E-01 | 0.905 |
| Coronavirus Pathogenesis Pathway | 2.52E+01 | 2.91E-01 | 2.517 |
| FAT10 Signaling Pathway | 2.47E+01 | 5.71E-01 | 1 |
| Mitochondrial Dysfunction | 1.99E+01 | 2.81E-01 | NaN |
| Huntington's Disease Signaling | 1.94E+01 | 2.19E-01 | -2.5 |
| Oxidative Phosphorylation | 1.83E+01 | 3.33E-01 | 0.164 |
| Regulation of eIF4 and p70S6K Signaling | 1.82E+01 | 2.63E-01 | -0.632 |
| Sirtuin Signaling Pathway | 1.80E+01 | 2.09E-01 | 0.905 |
| mTOR Signaling | 1.58E+01 | 2.26E-01 | -0.5 |
| Inhibition of ARE-Mediated mRNA Degradation Pathway | 1.54E+01 | 2.55E-01 | 0 |
| Protein Ubiquitination Pathway | 1.54E+01 | 2.00E-01 | NaN |
| Role of Hypercytokinemia/hyperchemokinemia in the Pathogenesis of Influenza | 1.46E+01 | 3.37E-01 | 5.014 |
| Role of PKR in Interferon Induction and Antiviral Response | 1.42E+01 | 2.65E-01 | 2.414 |
| Interferon Signaling | 1.28E+01 | 5.00E-01 | 3.153 |
| Glucocorticoid Receptor Signaling | 1.15E+01 | 1.34E-01 | NaN |
| TREM1 Signaling | 1.14E+01 | 3.12E-01 | 3.838 |
| Estrogen Receptor Signaling | 1.06E+01 | 1.47E-01 | 0.801 |
| Production of Nitric Oxide and Reactive Oxygen Species in Macrophages | 1.02E+01 | 1.94E-01 | 2.959 |
| NRF2-mediated Oxidative Stress Response | 9.05E+00 | 1.69E-01 | 0.408 |
| Unfolded protein response | 9.00E+00 | 2.56E-01 | 1.807 |
| Acute Phase Response Signaling | 8.77E+00 | 1.84E-01 | 2.353 |
| Activation of IRF by Cytosolic Pattern Recognition Receptors | 8.61E+00 | 2.92E-01 | 1.698 |
| HIF1Œ± Signaling | 8.51E+00 | 1.73E-01 | 1.029 |
| iNOS Signaling | 8.42E+00 | 3.40E-01 | 2.309 |
| Death Receptor Signaling | 8.41E+00 | 2.40E-01 | 2.065 |
| Hepatic Fibrosis Signaling Pathway | 8.28E+00 | 1.33E-01 | 1.54 |
| LXR/RXR Activation | 8.17E+00 | 2.11E-01 | -0.229 |
| Ferroptosis Signaling Pathway | 8.13E+00 | 2.05E-01 | -0.392 |
| Neuroinflammation Signaling Pathway | 8.11E+00 | 1.45E-01 | 4.111 |

**<u>Supplemental Table 2</u>: Cellular networks impacted by SARS-CoV-2 infection within Dendritic Cells in unvaccinated animals (day 2 post challenge) relative to un-infected animals**

| Ingenuity Canonical Pathways | -log(p-value) | Ratio | z-score |
|---|---|---|---|
| EIF2 Signaling | 3.52E+01 | 4.20E-01 | -5.181 |
| Mitochondrial Dysfunction | 2.47E+01 | 4.04E-01 | NaN |
| Oxidative Phosphorylation | 2.06E+01 | 4.50E-01 | 2.263 |
| Huntington's Disease Signaling | 1.84E+01 | 2.90E-01 | -0.6 |
| Regulation of eIF4 and p70S6K Signaling | 1.76E+01 | 3.41E-01 | -2.138 |
| FAT10 Signaling Pathway | 1.74E+01 | 5.71E-01 | 0.447 |
| Coronavirus Pathogenesis Pathway | 1.71E+01 | 3.20E-01 | 0.126 |
| Protein Ubiquitination Pathway | 1.69E+01 | 2.84E-01 | NaN |
| BAG2 Signaling Pathway | 1.68E+01 | 4.64E-01 | 1.387 |
| Sirtuin Signaling Pathway | 1.64E+01 | 2.74E-01 | -0.651 |
| Inhibition of ARE-Mediated mRNA Degradation Pathway | 1.53E+01 | 3.35E-01 | -0.756 |
| mTOR Signaling | 1.31E+01 | 2.83E-01 | -0.816 |
| Estrogen Receptor Signaling | 1.21E+01 | 2.20E-01 | 1.432 |
| Role of PKR in Interferon Induction and Antiviral Response | 1.07E+01 | 3.09E-01 | 0 |
| CLEAR Signaling Pathway | 1.01E+01 | 2.32E-01 | 1.364 |
| Phagosome Maturation | 1.00E+01 | 2.83E-01 | NaN |
| Glucocorticoid Receptor Signaling | 9.26E+00 | 1.84E-01 | NaN |
| PI3K/AKT Signaling | 9.06E+00 | 2.51E-01 | -0.354 |
| Interferon Signaling | 8.77E+00 | 5.00E-01 | 3.153 |
| Unfolded protein response | 8.76E+00 | 3.33E-01 | 1.698 |
| Insulin Secretion Signaling Pathway | 8.70E+00 | 2.23E-01 | 4.648 |
| Role of Hypercytokinemia/hyperchemokinemia in the Pathogenesis of Influenza | 8.62E+00 | 3.37E-01 | 2.785 |
| Production of Nitric Oxide and Reactive Oxygen Species in Macrophages | 7.78E+00 | 2.41E-01 | -1.372 |
| FcŒ≥ Receptor-mediated Phagocytosis in Macrophages and Monocytes | 7.63E+00 | 3.09E-01 | 0.186 |
| HER-2 Signaling in Breast Cancer | 7.47E+00 | 2.25E-01 | 0.295 |
| Hypoxia Signaling in the Cardiovascular System | 7.28E+00 | 3.29E-01 | 1 |
| Toll-like Receptor Signaling | 7.03E+00 | 3.21E-01 | 0 |
| ERK/MAPK Signaling | 6.92E+00 | 2.22E-01 | -0.48 |
| Actin Cytoskeleton Signaling | 6.76E+00 | 2.12E-01 | -0.926 |
| Necroptosis Signaling Pathway | 6.61E+00 | 2.42E-01 | 2.596 |

**Supplemental Table 3: Cellular networks impacted by SARS-CoV-2 infection within lymphocytes in unvaccinated animals (day 2 post challenge) relative to un-infected animals**

| Ingenuity Canonical Pathways | -log(p-value) | Ratio | z-score |
|---|---|---|---|
| EIF2 Signaling | 2.72E+01 | 2.99E-01 | -3.244 |
| FAT10 Signaling Pathway | 2.49E+01 | 5.89E-01 | 0 |
| BAG2 Signaling Pathway | 2.41E+01 | 4.64E-01 | 0 |
| Inhibition of ARE-Mediated mRNA Degradation Pathway | 1.88E+01 | 2.92E-01 | -2.132 |
| Coronavirus Pathogenesis Pathway | 1.86E+01 | 2.61E-01 | 0.42 |
| Huntington's Disease Signaling | 1.69E+01 | 2.16E-01 | -0.832 |
| Mitochondrial Dysfunction | 1.61E+01 | 2.63E-01 | NaN |
| Sirtuin Signaling Pathway | 1.56E+01 | 2.05E-01 | 1.605 |
| Oxidative Phosphorylation | 1.53E+01 | 3.15E-01 | -0.845 |
| Protein Ubiquitination Pathway | 1.50E+01 | 2.07E-01 | NaN |
| Death Receptor Signaling | 1.39E+01 | 3.23E-01 | 1.347 |
| PI3K Signaling in B Lymphocytes | 1.17E+01 | 2.45E-01 | 2.414 |
| Glucocorticoid Receptor Signaling | 1.13E+01 | 1.41E-01 | NaN |
| Role of PKR in Interferon Induction and Antiviral Response | 1.02E+01 | 2.35E-01 | 1.134 |
| Regulation of eIF4 and p70S6K Signaling | 9.96E+00 | 2.07E-01 | -1.897 |
| Interferon Signaling | 9.92E+00 | 4.44E-01 | 2.84 |
| Apoptosis Signaling | 9.77E+00 | 2.60E-01 | 0 |
| Activation of IRF by Cytosolic Pattern Recognition Receptors | 9.67E+00 | 3.23E-01 | 1.606 |
| Estrogen Receptor Signaling | 9.11E+00 | 1.47E-01 | 0.667 |
| Role of Hypercytokinemia/hyperchemokinemia in the Pathogenesis of Influenza | 8.70E+00 | 2.67E-01 | 3.962 |
| Natural Killer Cell Signaling | 8.61E+00 | 1.86E-01 | 1.029 |
| Primary Immunodeficiency Signaling | 8.34E+00 | 3.21E-01 | NaN |
| JAK/STAT Signaling | 7.64E+00 | 2.56E-01 | 1.606 |
| TNFR2 Signaling | 7.59E+00 | 4.06E-01 | 1.265 |
| Production of Nitric Oxide and Reactive Oxygen Species in Macrophages | 7.47E+00 | 1.78E-01 | 1.512 |
| mTOR Signaling | 7.33E+00 | 1.70E-01 | -1.155 |
| HER-2 Signaling in Breast Cancer | 7.03E+00 | 1.63E-01 | 2 |
| Sumoylation Pathway | 6.46E+00 | 2.14E-01 | 0.258 |
| Induction of Apoptosis by HIV1 | 6.45E+00 | 2.62E-01 | -1.069 |
| Ferroptosis Signaling Pathway | 6.19E+00 | 1.89E-01 | -0.816 |

## Supplemental Table 4: Cellular networks impacted by SARS-CoV-2 infection within epithelial cells in unvaccinated animals (day 2 post challenge) relative to un-infected animals

| Ingenuity Canonical Pathways | -log(p-value) | Ratio | z-score |
|---|---|---|---|
| EIF2 Signaling | 2.79E+01 | 2.46E-01 | -4.596 |
| Coronavirus Pathogenesis Pathway | 1.37E+01 | 1.77E-01 | 0.667 |
| Protein Ubiquitination Pathway | 1.03E+01 | 1.35E-01 | NaN |
| Oxidative Phosphorylation | 8.79E+00 | 1.89E-01 | -0.218 |
| Interferon Signaling | 8.23E+00 | 3.33E-01 | 3.317 |
| Mitochondrial Dysfunction | 7.91E+00 | 1.46E-01 | NaN |
| BAG2 Signaling Pathway | 7.70E+00 | 2.02E-01 | 1.414 |
| Role of Hypercytokinemia/hyperchemokinemia in the Pathogenesis of Influenza | 7.54E+00 | 1.98E-01 | 3.638 |
| Sirtuin Signaling Pathway | 7.36E+00 | 1.13E-01 | -0.6 |
| Regulation of eIF4 and p70S6K Signaling | 6.89E+00 | 1.34E-01 | NaN |
| FAT10 Signaling Pathway | 6.77E+00 | 2.32E-01 | NaN |
| Huntington's Disease Signaling | 6.65E+00 | 1.10E-01 | -1.414 |
| mTOR Signaling | 6.63E+00 | 1.23E-01 | -0.816 |
| Role of PKR in Interferon Induction and Antiviral Response | 5.86E+00 | 1.40E-01 | 0.688 |
| Unfolded protein response | 5.73E+00 | 1.67E-01 | 3.317 |
| Activation of IRF by Cytosolic Pattern Recognition Receptors | 5.18E+00 | 1.85E-01 | 1.155 |
| Antigen Presentation Pathway | 4.84E+00 | 2.31E-01 | NaN |
| Glucocorticoid Receptor Signaling | 4.03E+00 | 7.23E-02 | NaN |
| Putrescine Degradation III | 3.83E+00 | 2.73E-01 | -2.449 |
| NRF2-mediated Oxidative Stress Response | 3.82E+00 | 9.28E-02 | 0.258 |
| Aryl Hydrocarbon Receptor Signaling | 3.80E+00 | 1.07E-01 | 0.632 |
| Telomere Extension by Telomerase | 3.72E+00 | 3.33E-01 | NaN |
| Coronavirus Replication Pathway | 3.52E+00 | 1.78E-01 | 0.707 |
| Complement System | 3.31E+00 | 1.89E-01 | -0.378 |
| Inhibition of ARE-Mediated mRNA Degradation Pathway | 3.25E+00 | 9.94E-02 | 0.378 |
| Aldosterone Signaling in Epithelial Cells | 3.17E+00 | 9.76E-02 | NaN |
| Hypoxia Signaling in the Cardiovascular System | 3.16E+00 | 1.32E-01 | 2 |
| Phagosome Maturation | 2.86E+00 | 9.43E-02 | NaN |
| Estrogen Receptor Signaling | 2.84E+00 | 7.09E-02 | 0.243 |
| Clathrin-mediated Endocytosis Signaling | 2.83E+00 | 8.81E-02 | NaN |

**<u>Supplemental Table 5:</u> Cellular networks impacted by SARS-CoV-2 infection within myeloid cells in unvaccinated animals (day 2 post challenge) relative to vaccinated animals (day 2 post challenge**

| Ingenuity Canonical Pathways | -log(p-value) | Ratio | z-score |
|---|---|---|---|
| EIF2 Signaling | 4.75E+01 | 3.30E-01 | -5.154 |
| Coronavirus Pathogenesis Pathway | 2.46E+01 | 2.41E-01 | 3.464 |
| Regulation of eIF4 and p70S6K Signaling | 1.71E+01 | 2.12E-01 | NaN |
| mTOR Signaling | 1.53E+01 | 1.84E-01 | 0 |
| FAT10 Signaling Pathway | 1.51E+01 | 3.75E-01 | 1 |
| BAG2 Signaling Pathway | 1.41E+01 | 2.86E-01 | 0 |
| Role of Hypercytokinemia/hyperchemokinemia in the Pathogenesis of Influenza | 1.29E+01 | 2.67E-01 | 4.796 |
| Interferon Signaling | 1.18E+01 | 4.17E-01 | 3.742 |
| Huntington's Disease Signaling | 1.05E+01 | 1.34E-01 | -1.604 |
| Mitochondrial Dysfunction | 9.94E+00 | 1.64E-01 | NaN |
| Protein Ubiquitination Pathway | 9.12E+00 | 1.27E-01 | NaN |
| Inhibition of ARE-Mediated mRNA Degradation Pathway | 8.45E+00 | 1.55E-01 | 2.333 |
| Sirtuin Signaling Pathway | 8.42E+00 | 1.20E-01 | 0.186 |
| Atherosclerosis Signaling | 8.16E+00 | 1.68E-01 | NaN |
| Role of Pattern Recognition Receptors in Recognition of Bacteria and Viruses | 8.06E+00 | 1.54E-01 | 3.207 |
| Oxidative Phosphorylation | 8.02E+00 | 1.80E-01 | -2.236 |
| Role of PKR in Interferon Induction and Antiviral Response | 7.85E+00 | 1.62E-01 | 2.837 |
| Activation of IRF by Cytosolic Pattern Recognition Receptors | 7.67E+00 | 2.31E-01 | 1.807 |
| Necroptosis Signaling Pathway | 7.34E+00 | 1.46E-01 | 3.128 |
| LXR/RXR Activation | 7.23E+00 | 1.63E-01 | 0 |
| Acute Phase Response Signaling | 7.21E+00 | 1.35E-01 | 2.524 |
| Granulocyte Adhesion and Diapedesis | 7.03E+00 | 1.32E-01 | NaN |
| Death Receptor Signaling | 6.80E+00 | 1.77E-01 | 1 |
| TREM1 Signaling | 6.63E+00 | 1.95E-01 | 3.873 |
| Phagosome Maturation | 6.61E+00 | 1.38E-01 | NaN |
| Pathogenesis of Multiple Sclerosis | 6.59E+00 | 6.67E-01 | NaN |
| Iron homeostasis signaling pathway | 6.34E+00 | 1.44E-01 | NaN |
| HIF1Œ± Signaling | 6.22E+00 | 1.20E-01 | 2.2 |
| Neuroinflammation Signaling Pathway | 6.03E+00 | 1.01E-01 | 4.017 |
| Complement System | 6.00E+00 | 2.70E-01 | 0.707 |

**<u>Supplemental Table 6</u>: Cellular networks impacted by SARS-CoV-2 infection within dendritic cells in unvaccinated animals (day 2 post challenge) relative to vaccinated animals (day 2 post challenge**

| Ingenuity Canonical Pathways | -log(p-value) | Ratio | z-score |
|---|---|---|---|
| EIF2 Signaling | 4.51E+01 | 4.29E-01 | -4.808 |
| Regulation of eIF4 and p70S6K Signaling | 2.33E+01 | 3.46E-01 | -2.309 |
| Mitochondrial Dysfunction | 2.29E+01 | 3.51E-01 | NaN |
| Coronavirus Pathogenesis Pathway | 2.15E+01 | 3.15E-01 | 0.508 |
| Oxidative Phosphorylation | 2.12E+01 | 4.14E-01 | 2.236 |
| Huntington's Disease Signaling | 1.93E+01 | 2.61E-01 | -0.962 |
| BAG2 Signaling Pathway | 1.82E+01 | 4.40E-01 | 0 |
| FAT10 Signaling Pathway | 1.68E+01 | 5.18E-01 | 0.447 |
| Sirtuin Signaling Pathway | 1.61E+01 | 2.40E-01 | 0.143 |
| mTOR Signaling | 1.56E+01 | 2.69E-01 | -1.147 |
| Protein Ubiquitination Pathway | 1.47E+01 | 2.36E-01 | NaN |
| Estrogen Receptor Signaling | 1.26E+01 | 1.93E-01 | 0 |
| TREM1 Signaling | 1.15E+01 | 3.64E-01 | -1.177 |
| Inhibition of ARE-Mediated mRNA Degradation Pathway | 1.12E+01 | 2.61E-01 | -0.688 |
| Glucocorticoid Receptor Signaling | 1.09E+01 | 1.65E-01 | NaN |
| Role of Hypercytokinemia/hyperchemokinemia in the Pathogenesis of Influenza | 1.09E+01 | 3.37E-01 | 2.414 |
| Role of PKR in Interferon Induction and Antiviral Response | 9.92E+00 | 2.65E-01 | 0.539 |
| Insulin Secretion Signaling Pathway | 9.67E+00 | 2.01E-01 | 2.722 |
| Interferon Signaling | 9.32E+00 | 4.72E-01 | 2.5 |
| Production of Nitric Oxide and Reactive Oxygen Species in Macrophages | 9.26E+00 | 2.25E-01 | -1.808 |
| PI3K/AKT Signaling | 9.19E+00 | 2.21E-01 | -1.225 |
| Toll-like Receptor Signaling | 8.97E+00 | 3.21E-01 | 0.535 |
| Neuroinflammation Signaling Pathway | 8.92E+00 | 1.86E-01 | -0.42 |
| HER-2 Signaling in Breast Cancer | 8.77E+00 | 2.07E-01 | -0.447 |
| Necroptosis Signaling Pathway | 8.64E+00 | 2.36E-01 | 1.808 |
| Signaling by Rho Family GTPases | 8.58E+00 | 1.94E-01 | -1.859 |
| RAC Signaling | 8.53E+00 | 2.46E-01 | -2.2 |
| Hypoxia Signaling in the Cardiovascular System | 8.50E+00 | 3.16E-01 | -1.134 |
| Activation of IRF by Cytosolic Pattern Recognition Receptors | 7.72E+00 | 3.23E-01 | 1.606 |
| Induction of Apoptosis by HIV1 | 7.72E+00 | 3.23E-01 | 0.471 |

**Supplemental Table 7: Cellular networks impacted by SARS-CoV-2 infection within lymphocytes in unvaccinated animals (day 2 post challenge) relative to vaccinated animals (day 2 post challenge**

| Ingenuity Canonical Pathways | -log(p-value) | Ratio | z-score |
|---|---|---|---|
| Coronavirus Pathogenesis Pathway | 1.49E+01 | 1.92E-01 | 0.164 |
| FAT10 Signaling Pathway | 1.45E+01 | 3.75E-01 | 0.447 |
| EIF2 Signaling | 1.27E+01 | 1.70E-01 | -1.528 |
| Interferon Signaling | 1.26E+01 | 4.44E-01 | 2.84 |
| BAG2 Signaling Pathway | 1.15E+01 | 2.62E-01 | -2.646 |
| Role of Hypercytokinemia/hyperchemokinemia in the Pathogenesis of Influenza | 1.13E+01 | 2.56E-01 | 3.838 |
| Death Receptor Signaling | 1.11E+01 | 2.40E-01 | 0.853 |
| Sirtuin Signaling Pathway | 9.77E+00 | 1.34E-01 | 0.707 |
| Protein Ubiquitination Pathway | 9.39E+00 | 1.35E-01 | NaN |
| Huntington's Disease Signaling | 9.04E+00 | 1.31E-01 | -2.138 |
| Mitochondrial Dysfunction | 8.54E+00 | 1.58E-01 | NaN |
| Inhibition of ARE-Mediated mRNA Degradation Pathway | 8.46E+00 | 1.61E-01 | -0.302 |
| Oxidative Phosphorylation | 8.21E+00 | 1.89E-01 | -4.146 |
| Activation of IRF by Cytosolic Pattern Recognition Receptors | 8.12E+00 | 2.46E-01 | 1.5 |
| Primary Immunodeficiency Signaling | 7.28E+00 | 2.50E-01 | NaN |
| PI3K Signaling in B Lymphocytes | 6.88E+00 | 1.54E-01 | 2.183 |
| Antigen Presentation Pathway | 6.42E+00 | 2.82E-01 | NaN |
| Role of PKR in Interferon Induction and Antiviral Response | 5.99E+00 | 1.47E-01 | 2.236 |
| Crosstalk between Dendritic Cells and Natural Killer Cells | 5.96E+00 | 1.76E-01 | 1.604 |
| Glucocorticoid Receptor Signaling | 5.87E+00 | 8.61E-02 | NaN |
| Necroptosis Signaling Pathway | 5.57E+00 | 1.34E-01 | 2.4 |
| Phagosome Maturation | 5.48E+00 | 1.32E-01 | NaN |
| TREM1 Signaling | 5.47E+00 | 1.82E-01 | 2.138 |
| Natural Killer Cell Signaling | 5.46E+00 | 1.21E-01 | 1.043 |
| TNFR2 Signaling | 5.34E+00 | 2.81E-01 | -1.134 |
| TNFR1 Signaling | 5.08E+00 | 2.12E-01 | -1.897 |
| Role of Pattern Recognition Receptors in Recognition of Bacteria and Viruses | 5.06E+00 | 1.28E-01 | 1.508 |
| Neuroinflammation Signaling Pathway | 4.93E+00 | 9.78E-02 | 1.569 |
| Agranulocyte Adhesion and Diapedesis | 4.92E+00 | 1.12E-01 | NaN |
| NAD Signaling Pathway | 4.72E+00 | 1.26E-01 | 1 |

**<u>Supplemental Table 8:</u> Cellular networks impacted by SARS-CoV-2 infection within lymphocytes in unvaccinated animals (day 2 post challenge) relative to vaccinated animals (day 2 post challenge**

| Ingenuity Canonical Pathways | -log(p-value) | Ratio | z-score |
|---|---|---|---|
| EIF2 Signaling | 3.47E+01 | 2.77E-01 | -4.49 |
| Coronavirus Pathogenesis Pathway | 2.19E+01 | 2.27E-01 | 1.043 |
| Regulation of eIF4 and p70S6K Signaling | 1.71E+01 | 2.12E-01 | -2.236 |
| mTOR Signaling | 1.46E+01 | 1.79E-01 | -1.897 |
| Role of Hypercytokinemia/hyperchemokinemia in the Pathogenesis of Influenza | 8.35E+00 | 2.09E-01 | 2.828 |
| Actin Cytoskeleton Signaling | 8.09E+00 | 1.27E-01 | -1.8 |
| Interferon Signaling | 7.15E+00 | 3.06E-01 | 2.53 |
| Mitochondrial Dysfunction | 6.65E+00 | 1.35E-01 | NaN |
| Germ Cell-Sertoli Cell Junction Signaling | 6.65E+00 | 1.35E-01 | NaN |
| RAC Signaling | 5.76E+00 | 1.38E-01 | -2 |
| Remodeling of Epithelial Adherens Junctions | 5.73E+00 | 1.91E-01 | -1.633 |
| ILK Signaling | 5.45E+00 | 1.15E-01 | -1.789 |
| Ferroptosis Signaling Pathway | 5.43E+00 | 1.36E-01 | -1.414 |
| Sirtuin Signaling Pathway | 5.39E+00 | 9.93E-02 | 0.218 |
| Production of Nitric Oxide and Reactive Oxygen Species in Macrophages | 5.25E+00 | 1.15E-01 | -3.13 |
| Role of PKR in Interferon Induction and Antiviral Response | 5.24E+00 | 1.32E-01 | 0 |
| Complement System | 5.04E+00 | 2.43E-01 | -1.342 |
| Integrin Signaling | 4.99E+00 | 1.08E-01 | -1.606 |
| Leukotriene Biosynthesis | 4.90E+00 | 4.00E-01 | -0.447 |
| Signaling by Rho Family GTPases | 4.72E+00 | 9.70E-02 | -2.132 |
| Leukocyte Extravasation Signaling | 4.66E+00 | 1.09E-01 | -1.213 |
| Oxidative Phosphorylation | 4.58E+00 | 1.35E-01 | -1.807 |
| RHOGDI Signaling | 4.44E+00 | 1.02E-01 | 0.728 |
| Activation of IRF by Cytosolic Pattern Recognition Receptors | 4.42E+00 | 1.69E-01 | 0.905 |
| Atherosclerosis Signaling | 4.29E+00 | 1.22E-01 | NaN |
| NRF2-mediated Oxidative Stress Response | 4.25E+00 | 9.70E-02 | -1.941 |
| Sertoli Cell-Sertoli Cell Junction Signaling | 4.24E+00 | 1.02E-01 | NaN |
| Eicosanoid Signaling | 4.23E+00 | 1.62E-01 | 0.447 |
| IL-8 Signaling | 4.12E+00 | 1.00E-01 | -2.524 |
| Epithelial Adherens Junction Signaling | 3.86E+00 | 1.08E-01 | 0.243 |
